# Oxidative stress, a trigger of hepatitis C and B virus-induced liver carcinogenesis

**DOI:** 10.18632/oncotarget.13904

**Published:** 2016-12-11

**Authors:** Alexander V. Ivanov, Vladimir T. Valuev-Elliston, Daria A. Tyurina, Olga N. Ivanova, Sergey N. Kochetkov, Birke Bartosch, Maria G. Isaguliants

**Affiliations:** ^1^ Engelhardt Institute of Molecular Biology, Russian Academy of Sciences, Moscow, Russia; ^2^ Inserm U1052, Cancer Research Center Lyon, University of Lyon, Lyon, France; ^3^ DevWeCan Laboratories of Excellence Network, France; ^4^ Riga Stradins University, Riga, Latvia; ^5^ Department of Microbiology, Tumor and Cell Biology, Karolinska Institutet, Stockholm, Sweden

**Keywords:** hepatitis C virus, hepatitis B virus, reactive oxygen species, pathogenesis, carcinogenesis

## Abstract

Virally induced liver cancer usually evolves over long periods of time in the context of a strongly oxidative microenvironment, characterized by chronic liver inflammation and regeneration processes. They ultimately lead to oncogenic mutations in many cellular signaling cascades that drive cell growth and proliferation. Oxidative stress, induced by hepatitis viruses, therefore is one of the factors that drives the neoplastic transformation process in the liver. This review summarizes current knowledge on oxidative stress and oxidative stress responses induced by human hepatitis B and C viruses. It focuses on the molecular mechanisms by which these viruses activate cellular enzymes/systems that generate or scavenge reactive oxygen species (ROS) and control cellular redox homeostasis. The impact of an altered cellular redox homeostasis on the initiation and establishment of chronic viral infection, as well as on the course and outcome of liver fibrosis and hepatocarcinogenesis will be discussed The review neither discusses reactive nitrogen species, although their metabolism is interferes with that of ROS, nor antioxidants as potential therapeutic remedies against viral infections, both subjects meriting an independent review.

## INTRODUCTION

According to World Health Organization, non-communicative diseases are responsible for almost 68% deaths worldwide, and various types of cancer comprise the second most significant group of diseases [1, 2]. The main contributors to human mortality are lung, stomach, colorectal, breast, and liver cancer. The GLOBOCAN data from International Agency for Research on Cancer (IARC) estimated 14.1 mln new cases of cancer and 8.2 mln cancer-related deaths worldwide in 2012 [3, 4]. Death rates for all cancers combined among men and women of all major racial and ethnic groups and in most cancer sites are declining by 1.5% per year [5]. However, studies of the Centers for Disease Control and Prevention, the American Cancer Society, the National Cancer Institute, and the North American Association of Central Cancer Registries demonstrated that the incidence of liver cancer is increasing at a rapid rate of 2.3 percent per year (from 2003 to 2012), and the rate of deaths due to liver cancer is growing faster than for any other type of cancer [5]. Liver cancer is the fifth most common cancer among men, the ninth most common among women, and the second most common cause of cancer death for men and women combined [3, 6]. In 2012 a number of new cases of liver cancer and deaths related to it were estimated as 782,500 and 745,000, respectively [3, 4]. Hepatocellular carcinoma (HCC) is the most common (70-90%) histologic type of primary liver cancer [3]. The global age-standardized incident rate of hepatocellular carcinoma in men and women is 15.3 and 5.3 per 100,000, respectively thus forming a mean value of 10.1 per 100,000 general population [7]. The age-standardized incidence of HCC is highest in East Asia and South Africa [7]. Indeed, almost half of hepatocellular carcinoma incidence and deaths are attributed to China [3, 4].

Various types of cancer are triggered by bacterial and viral infections. According to 2012 GLOBOCAN statistics, 2.2 mln out of 14.1 mln are attributable to infections [8]. Among them are chronic infections with hepatitis B and C viruses (HBV, HCV) that are well documented risk factors for HCC. Globally, both infections are reported to contribute to greater than ca. 80% of HCC cases [5, 8, 9]. In developing countries they account for >90% of all HCC cases, whereas in developed countries - for 40% [7]. Comparing HCC incidence rates due to viral infections versus other etiologies revealed that an increase of HBV or HCV prevalence by 1% elevates by 14% and 10%, respectively, the incidence of liver cancer [7]. HCC incidence has severely increased in Western Europe and Northern America, *i.e.* the regions that initially had low prevalence of this type of cancer. This increase is especially dramatic in the USA: between 1975 and 2011 the population adjusted incidence rose more than three-fold [3, 4]. In contrast, HCC incidence tends to decrease in regions with historically high rates such as China and Japan, probably due to reduction in HCV and HBV prevalence [3, 4]. The Global Burden of Disease Study 2013 (GBD 2013) and HALE Collaborators described the input of liver cancer in life shortening and reduction of normal life as an index of disability-adjusted life-years (DALYs) [10]. DALYs were calculated as a sum of years of life lost due to premature mortality (YLLs) and years lived with disability (YLDs). DALYs for liver cancer increased by 9.2% from 2005 to 2013. Hepatitis B and C viruses accounted for 41% and 38% of all DALYs due to liver cancer. Moreover, DALYs due to HBV- and HCV-induced liver cancer increased by 4.8% and 35.1% from 2005 to 2013. These data depict the huge problem, which hepatitis B and C pose to our healthcare systems.

Both HBV and HCV establish chronic infection of the liver characterized by persistent inflammation that stimulates regenerative liver fibrosis and ultimately cirrhosis. At advanced stages of fibrosis, the risk of HCC incidence increases considerably. HCV RNA-positive patients have a higher risk of HCC and death from HCC than HCV RNA-negative patients [11–14]. Similarly, elevated HBV DNA levels, alanine aminotransferase (ALT) levels, and hepatitis B virus envelope antigen (HBeAg) status are among the most important determinants of risk of progression to cirrhosis, whereas HBV DNA levels (>2,000 IU/mL), HBeAg status, and cirrhosis are the key predictors of HCC incidence [15]. These facts suggest that chronic viral replication is a key element in hepatitis virus induced carcinogenicity. However, patients spontaneously clearing HCV infection remain at an elevated risk of developing HCC with a 4.71-fold lower rate than chronic patients [11]. The latter indicates the carcinogenic potential of not only chronic, but also of a time-limited viral replication. With the arrival of direct acting antivirals for cure of hepatitis C, it is becoming clear that fibrosis and even cirrhosis are reversible. However, this is not the case in all patients, and raises the important question to what extent the pro-carcinogenic actions induced by hepatitis viruses can persist upon viral elimination. Answers to these questions will be vital for the development of efficient treatment modalities and priorisation of patients for treatment access.

HCV and HBV driven hepatocarcinogenesis is multifactorial, but a key factor underlying the oncogenic effects of HCV and HBV, as well as single viral proteins, is their capacity to induce oxidative stress [16, 17]. Liver regeneration / fibrosis in the context of an oxidative and inflammatory microenvironment is likely the driving force. Here, we comprehensively review the molecular mechanisms by which hepatitis B and C viruses induce oxidative stress and trigger ROS sensitive signaling cascades and inflammatory processes that predispose to cancer.

## REACTIVE OXYGEN SPECIES, THEIR GENERATION AND NEUTRALIZATION

**Figure 1:**
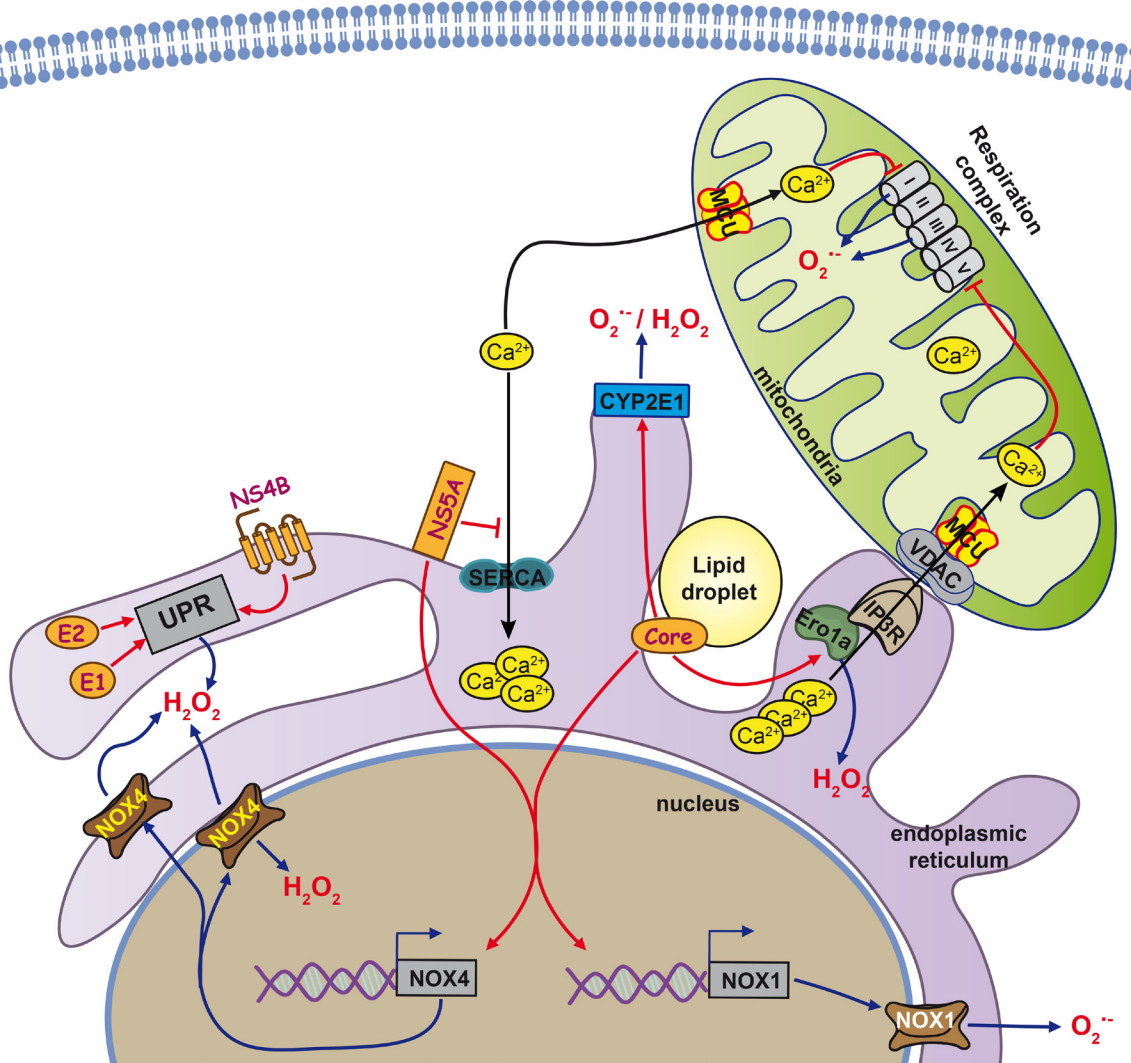
Cellular sources of reactive oxygen species in HCV-infected cells The predominant forms of reactive oxygen species (ROS) in the cell are superoxide anion (O_2_^•−^) and hydrogen peroxide (H_2_O_2_), but other forms exist (see text for more details). Major sources for O_2_^•−^are the electron transport chain in mitochondria, transmembrane NADPH oxidases (Nox) or cytochromes P450 (CYP). Dismutation of O_2_^•−^ leads to the formation of H_2_O_2._Other major sources of H_2_O_2_ are catabolic reactions or formation of disulfide bonds during protein folding involving enzymes such as endoplasmic reticulum oxidoreductin-1alpha (Ero1alpha). Key events in the cell that are associated with increases in ROS production are e.g. the unfolded protein response (UPR) at the ER, triggered directly by several HCV proteins such as the glycoproteins E1 and E2 as well as non-structural protein NS4B. Furthermore, in response to stress signals, Ca2+ is released from the ER and taken up by mitochondria via the mitochondrial calcium uniporter (MCU). Ca2+ uptake by MCU can also occur in the context of mitochondria associated membranes, points of contact between ER and mitochondria structurally formed by inositol triphosphate repector (IP3R) and Voltage dependent anion channel protein (VDAC). In the mitochondria, Ca levels directly impact the functioning of the electron transport chain and can increase ROS production.

Reactive oxygen species (ROS) are highly reactive oxygen intermediates that can modify various biological molecules, thus posing a threat to the living cell. ROS comprise superoxide anion (O_2_^•−^), hydroxyl radical (HO^•^), singlet oxygen (^1^O_2_), hydrogen peroxide (H_2_O_2_) and other types of compounds/intermediates [18, 19]. They are formed in the cell during many physiological processes, such as mitochondrial oxidative phosphorylation, protein folding in the endoplasmic reticulum (ER), catabolism of various classes of endogenous molecules, such as lipids, biogenic polyamines and amino acids, or exogenously introduced substances such as drugs (Figure 1). Superoxide anion is mainly produced as a result of electron escape from the mitochondrial electron transport chain, transmembrane NADPH oxidases (NOX), cytochromes P450 (CYP), *etc*. Hydrogen peroxide is mainly formed as a stoichiometric by-product in the catabolic reactions, as well as in the formation of disulfide bonds during protein folding. Hydroxyl radical is produced in the Fenton reaction of decomposition of hydrogen peroxide in the presence of divalent iron cations:

Fe^2+^ + H_2_O_2_ → Fe^3+^ + HO^•^ + HO^−^

or the Haber-Weiss reaction:

O_2_^•−^+H_2_O_2_ → HO^•^ + O_2_ + HO^−^

Different types of ROS have different stabilities and display different oxidizing capacities towards other molecules. The most reactive ROS is the hydroxyl radical HO^•^, characterized by a half-life of app 10^−9^ s [18]. Unlike superoxide and hydrogen peroxide, it is not eliminated by the enzymatic reaction, but only scavenged by antioxidants. If not scavenged, it reacts directly at the production site, oxidizing biological molecules in the immediate vicinity. HO^•^ induces formation of single and more complex damage in cellular DNA such as tandem lesions, intra- and interstrand cross-links, and DNA-protein cross-links resulting from one radical hit; disrupt disulfide bonds in proteins, such as fibrinogen, resulting in their unfolding and scrambled refolding into abnormal spatial configurations, and causes lipid peroxidation [20, 21]. Superoxide itself has relatively low oxidizing capacity due to its charge that restricts interaction with electron-rich moieties. However it could either react with NO to form peroxynitrite or be protonated, and the resulting perhydrohyl radical to act as a strong prooxidant [22]. Finally, another ROS, H_2_O_2,_ in contrast to O_2_^•−^and HO^•^, is relatively stable (cellular half-life ~1 ms, steady-state levels ~10^−7^ M) [23]. Due to a selective reactivity and long half-life, H_2_O_2_ is cell permeable and diffuses away from the sites of its production, in contrast to O_2_^•−^ and other short-lived ROS that are restricted to a sub-cellular volume surrounding the site of their generation [23, 24]. Noteworthy, it is able to trigger cell-signaling cascades [25].

Eukaryotic cells have developed a special antioxidant defense system that counteracts the hazardous effects of ROS. The system is comprised of the low molecular weight antioxidants (including glutathione, α-tocopherol, vitamin C, etc.) [26] and several types of ROS-scavenging enzymes referred to as phase II enzymes [27]. Neutralization of O_2_^•−^ is achieved by SOD which converts it into H_2_O_2_ and inhibits the formation of clastogenic factors [28]. Mammalian cells encode three isoforms of this enzyme. Cu/Zn-SOD (SOD1) is expressed mainly in the cytoplasm and MnSOD (SOD2) is found in the mitochondrial matrix, whereas EC-SOD (SOD3) represents an extracellular enzyme. H_2_O_2_ is neutralized by several families of enzymes, including catalase (CAT), peroxiredoxins (Prdx) 1-6 [29], and glutathione peroxidases (GPx) 1-8 [30], represented by specific isoforms with different cellular localizations. Of these enzymes, GPx4 is unique in its ability to scavenge lipid peroxides. Peroxiredoxins and glutathione peroxidases catalyze the scavenging reaction extremely efficiently [22]. Low molecular weight compounds termed antioxidants can under certain circumstances scavenge ROS; however, the kinetics of these reactions is generally very slow and not decisive for the actual levels of ROS in the cell [31]. Due to this, the actual levels of ROS are not uniform throughout the cell; instead, they strongly depend on the levels of ROS-producing enzymes and the existence of scavenging enzyme in close proximity to the site of ROS generation [32].

Expression of a wide range of phase II enzymes, as well as of the enzymes involved in the biosynthesis of glutathione and its maintenance or recycling in a the reduced form, is controlled by NF-E2-related factor 2 (Nrf2) [27]. The control is executed by Nrf2 binding to the common sequences within the promoters of the responsive genes, and the respective regions referred to as Antioxidant Response Elements (ARE) [27]. In the absence of the oxidative stress, Nrf2 is retained in the cytoplasm by Keap1 protein. Elevation of ROS levels leads to phosphorylation of Nrf2, disruption of this interaction and subsequent translocation of the transcription factor to the nucleus.

Carcinogenesis is characterized by dysregulation of various ROS-producing and ROS-scavenging enzymes. Eventually all above mentioned ROS-producing enzymes were implicated in either direct promotion of tumor formation, or in activation of procarcinogens into carcinogens. It all may result in genomic instability and increased mutation rate. Activation of the defense pathways may lead to partial resistance towards cytotoxic effect of ROS thus allowing cells to survive the oxidative stress. Finally, Nrf2/ARE pathway may also contribute to metabolic adaptation of the transformed cell (such as glycolytic adaptation) and to anticancer drug resistance. Investigation of ROS production and ROS-scavenging processes in cells infected with HBV or HCV can shed light on actual mechanisms of carcinogenesis.

## HEPATITIS C VIRUS

### HCV biology

Worldwide, over 150 million people are chronic carriers of hepatitis C virus (HCV) [33]. In the majority (up to 80%) of acute hepatitis C cases, the virus cannot be cleared and develops into chronic hepatitis C (CHC), characterized by chronic liver inflammation and fibrosis [34] and an increased risk of the development of liver cirrhosis and hepatocellular carcinoma [35]. The disease is aggravated and accelerated by the metabolic alterations, such as insulin resistance, steatosis and iron overload [36]. Extrahepatic manifestations are also frequently observed in chronic HCV carriers including amongst others non-Hodgkin lymphoma (NHL) (for example, see [11]) and in particular diffuse large cell NHL [37, 38] as well as mixed cryoglobulinemia [39, 40].

HCV belongs to the *Flaviviridae* family [35]. Its genome is constituted by a (+)-strand RNA of approximately 9.6 kb, with a single open reading frame flanked by 5′- and 3′-untranslated regions (UTRs). Translation of the genome and subsequent processing of the synthesized polypeptide chain results in the production of ten protein products. Three, nucleocapsid (core) and E1 and E2 glycoproteins, are referred to as structural, and the rest - NS2 or p7 (forms ion channels), proteases NS2 and NS3, NS4A (protease cofactor), NS4B (transmembrane protein located in endoplasmic reticulum (ER)), NS5A (regulatory phosphoprotein) and NS5B (RNA-dependent RNA polymerase) - as nonstructural (NS).

HCV is internalized by concordant action of a set of at least four cell receptors: CD81, SR-BI, claudin, and occludin. Internalization is followed by the clathrin-dependent endocytosis [41]. Replication of the virus occurs on the ER outer membrane and ER-derived vesicles [35, 42]. Virions are produced as a result of genome encapsidation by core protein, with subsequent formation of the envelope from E1/E2 glycoproteins and lipids and lipoproteins of the host cell [43].

### Oxidative stress in HCV-infected patients

It has been clearly established that hepatitis C is associated with strong oxidative stress. This was revealed in liver tissues and in blood serum/plasma samples of CHC patients using a variety of techniques, including direct measurement of ROS, quantification of DNA, lipid and protein oxidation products, as well as by assessing the total oxidant/antioxidant status or the levels of individual antioxidants. Screening of the liver biopsies of chronic HCV carriers revealed a two to five log elevation of the levels of oxygen radicals [44, 45], and stress markers such as 7,8-dihydro-8-oxoguanine (8-oxoG) [46], malondialdehyde (MDA) [47, 48] and 4-hydroxynonenal- (HNE)- and other protein adducts [49, 50]. Serum/plasma of such patients is characterized by the increased levels of a wide array of oxidative stress markers such as MDA [51–59], lipid peroxides [57], protein carbonyl content [51], oxysterols [60], and thioredoxin [61, 62]. A similar increase in 8-oxoG and lipid peroxidation products was observed in their peripheral blood mononuclear cells (PBMCs) [55, 63, 64], and of some markers, such as 8-isoprostane, even in the urine [65]. Sera of CHC patients also exhibit an increased level of clastogenic factors [53, 54]. Due to this, DNA of leukocytes from chronic hepatitis C virus carriers contains up to 20 times more apurine/apyrimidine sites than DNA of healthy individuals [66]. Cells of CHC patients are also characterized by a decrease in the ratio between mitochondrial and nuclear DNA, which indicates an increased ROS production in mitochondria [67]. In most (though not all [68]), cases the levels of oxidative stress markers in patient sera/plasma strongly correlate with their hepatic levels and with the general redox status [47]. Furthermore, there is mounting evidence that HCV can also deregulate the damage response to single- and double-stranded DNA lesions [69]. Interestingly, an increase in the levels of single-stranded DNA damage has been observed by Higgs *et al.* in mice transgenic for the entire complement of HCV proteins [70]. The latter implies that DNA damage does not require viral replication/propagation, but can also be induced by the (cumulative) activity of viral proteins.

Another feature of the oxidative stress in CHC patients is a decreased antioxidant capacity in liver and blood. Indeed, CHC patients often exhibit reduced total glutathione levels and an increased ratio between its oxidized (GSSG) and reduced (GSH) forms in liver [48] and blood or sera [64, 65], decreased levels of vitamins C and E [65], and total antioxidant status [50, 57, 64, 65, 71, 72]. These changes are more pronounced in genotype 1b than in genotypes 2a, b and especially 3a [48], and can even be identified in the background of HIV-1 coinfection, which also induces a profound oxidative stress [59].

Markers of oxidative stress are typical to all forms of viral hepatitis C, acute, chronic, and as well as occult [66, 73]. Changes in ROS production and glutathione content are actually much stronger in the acute stage than in the chronic infection [73], while the redox dysregulation in the occult hepatitis C is milder than in “classical” CHC [66].

### Sources of ROS in infected cells

At present, six viral proteins are known to induce and/or augment the production of ROS, namely HCV core [74–80], E1 [74], E2 [74, 81], NS3 [79, 82], NS4B [74, 83], and NS5A [74, 80, 84] (Figure 1). HCV core is believed to have the highest prooxidant capacity [74]. However, oxidative stress in response to HCV core is observed later than in response to NS5A protein, at least in the settings of protein overexpression in eukaryotic cells [80].

HCV triggers oxidative stress by simultaneous induction of several ROS-producing pathways and enzymes. They include Ca^2+^-mediated mitochondrial dysfunction, NOX1, 2 and 4, as well as cytochrome P450 2E1 (CYP2E1) and ER oxidoreductin 1α (Ero1α). These pathways are not fully independent; some are interregulated or have common mediators. The best studied mechanisms of ROS production induced by HCV involve mitochondrial dysfunctions triggered by HCV core and presumably also by several other viral proteins. A general trigger for the dysfunction is localization of HCV core on the mitochondrial outer membrane [85], or matrix binding to heat shock protein 60 (Hsp60) [86], which induces a release of calcium ions from their storage in the ER and their subsequent accumulation in mitochondria [76, 87]. Influx of Ca^2+^ into mitochondria occurs through calcium uniporter (MCU) [76], a transporter localized at the inner membrane of mitochondria [88, 89]. A detailed analysis shows that HCV core is localized at the surface of mitochondria, ER or lipid droplets [90], and also concentrated on the mitochondria-associated membranes (MAM) [91], which constitute the contact sites between mitochondria and ER [92]. MAMs are the sites where calcium ions can be directly translocated from ER to mitochondria [89]. They are formed through interaction of inositol 1,4,5-triphosphate receptor (IP3R) on the ER membrane with voltage-dependent anion channel (VDAC) on the outer membrane of mitochondria. The process is controlled by Ero1α, which binds to IP3R [93]. Ero1α-dependent ER calcium leakage to mitochondria through MAMs is a critical event in the procaspase-mediated cell death; Ero1α silencing inhibits both calcium release from ER and cell death [94]. It was intriguing to find that HCV core can trigger a marked increase in the expression of Ero1α, whereas the inhibition of Ero1α induction was able to significantly abrogate the mitochondrial production of superoxide [95], defining the direct relation between the expression of HCV core and induction of apoptosis of the expressing cell.

Accumulation of calcium ions in mitochondria is believed to alter normal functioning of the respiratory chain. HCV core-expressing Huh7 cells and hepatocytes of mice transgenic for HCV core demonstrate a pronounced decrease in the activity of complex I [75, 87]. This activity is completely restored in the presence of the inhibitors of IP3R and MCU, thus supporting the role of calcium ions in HCV core-induced oxidative stress. Additional effects could arise from the binding of HCV core to prohibitin [96], a chaperone that has multiple functions in mitochondria and can bind to complex IV and complex I [97]. The mechanism by which calcium ions can trigger ROS production in HCV-infected cells has not been deciphered. To our knowledge, it has not been shown that Ca^2+^ can directly bind to and inhibit complex I activity. Jekabstone *et al.* reported that complex I can be inhibited only by a combination of Ca^2+^ and NO that leads to generation of peroxynitrite [98]. We cannot rule out that HCV and its core protein exploit this mechanism to inhibit complex I activity since they are known to induce NO synthase with concomitant production of NO, at least in non-liver cells [99]. Nevertheless, other calcium-dependent ROS-producing enzymes in mitochondria should be considered such as α-ketoglutarate dehydrogenase (α-KGDH) and pyruvate dehydrogenase (PHD) that are located in mitochondrial matrix [100, 101]. These enzymes are known to produce hydrogen peroxide as well as superoxide. In case of PHD ROS production is associated with dihydrolipoamide dehydrogenase that is the key subunit of the complex [102]. Both PDH and α-KGDH are known to be stimulated by calcium ions at micromolar concentrations [103, 104]. However, these enzymes have never been evaluated as possible HCV-induced sources of ROS.

The primary event that might trigger both calcium efflux from the ER and subsequent oxidative stress is ER stress and subsequent unfolded protein response (UPR). HCV core [105], as well as E1/E2 glycoproteins [106] and NS4B protein [83, 107], induce UPR and calcium efflux from the ER. UPR is a cellular program that mediates induction of components of the protein-folding machinery, activation of ER-associated protein degradation (ERAD), and, if stress is not resolved, proapoptotic events [108, 109]. The latter are induced by GADD153/CHOP protein that accumulates during UPR. In this context, it is noteworthy that the CHOP arm of the UPR enhances both Ero1α expression and IP3R activity [110].

There is considerable uncertainty about the interrelation of the altered calcium homeostasis and ROS production in the presence of NS5A protein. On one hand, a study by Gong *et al.* suggested that oxidative stress is induced by this protein through the efflux of calcium ions from ER causing mitochondrial dysfunction [84]. This efflux is due to passive leakage of the ions [111]. On the other hand, an independent study revealed that calcium alterations are not a cause but rather a consequence of the NS5A-induced oxidative stress [112]. In our hands [113] no decrease in ROS production was observed in NS5A-expressing cells treated with the cell permeable calcium chelator BAPTA-AM or with an MCU inhibitor, drugs that stop the influx and prevent (according to Gong *et. al.* [84]) the mitochondrial dysfunction. This suggests an independence of the NS5A-associated ROS production of calcium ion influx which supports the findings of Dionisio *et al.* [112].

A second important mechanism of HCV-induced ROS production involves the induction of NADPH oxidases 1 and 4. This was demonstrated in patients with chronic viral hepatitis C, *in vitro* in cells productively replicating HCV or expressing genomic or subgenomic HCV replicons, or individual viral proteins such as HCV core [95, 114, 115]. Our recent data show that both HCV core and NS5A proteins induce the expression of NOX1 and NOX4 [95, 113]. Induction of these enzymes is due to accumulation of transforming growth factor β1 (TGF-β1), which controls the transcription of both genes. In 2011, it was elegantly shown that the proinflammatory stimuli can lead to the induction of both NOX isoforms *via* the cascade TGFβ1→NOX1→COX2→NOX4, where COX2 is cyclooxygenase 2, an enzyme involved in the biosynthesis of prostaglandin E2 [116]. We have presented evidence of this regulation in cells expressing NS5A [113]. However, in HCV core-expressing cells, TGF-β1 induces an independent accumulation of NOX1 and NOX4 [74], which puts the cascade TGF-β1→NOX1→COX2→NOX4 under question. Another counter-argument against is a difference in the kinetics of accumulation of NOX1 and NOX4 in HCV-infected cells: NOX1 is accumulated shortly after the infection, whereas a pronounced increase in the expression of NOX4 occurs only after two weeks [115].

NOX4 is localized to multiple organelles in the cell, including the ER and nucleus [115, 117]. In fact, in HCV-infected cells, the most pronounced increase in the levels of NOX4 is observed in the nucleus, which results in the production of ROS in a close proximity to the genomic DNA [115]. Its main product, unlike those of the other NOX/DUOX enzymes, is hydrogen peroxide [118]. However, we and others have clearly observed that inhibition of the HCV/HCV core-triggered induction of NOX4 results in a marked reduction of superoxide levels [95, 114, 115]. Furthermore, NOX4 was shown to be the primary NADPH-dependent superoxide producing enzyme in the hepatic nuclei and ER [117]. This discrepancy can be explained by different settings of the above experiments, namely, a different ratio between peroxide and superoxide produced by NOX4 *in vitro* when taken as a recombinant protein versus NOX4 in the context of the living cell. Hence, so far, the actual contribution of NOX4 into the production of various types of ROS in HCV-infected cells is unclear.

The data on the ability of NS3 protein to trigger oxidative bursts are also scarce [79, 82]. In monocytes, cells that do not support HCV infection, NS3 causes elevation of calcium levels in the cytoplasm, leading to the activation of the phagocytic NADPH oxidase (NOX2) and massive production of superoxide anions [82]. The role of NS3 in triggering ROS production in hepatocytes remains controversial: elevation of ROS levels was observed by Pal *et al.* [79], whereas in our hands, NS3-expressing Huh7 cells demonstrated no signs of oxidative stress [74].

Data on the possible role of other ROS-producing enzymes in mediating HCV-induced oxidative stress are scarce and mostly restricted to CYP2E1. This enzyme is localized on the ER and, to some extent, on the mitochondrial membranes, where it participates in catabolism of both endogenous and exogenous compounds, with the concomitant production of both superoxide and hydrogen peroxide [119–121]. CYP2E1 mediates a minor pathway of ethanol catabolism in healthy individuals; however, its expression is enhanced in patients with heavy alcohol consumption, in whom this pathway turns into the major route of ethanol detoxification, significantly contributing to the liver pathology [121]. Importantly, CYP2E1 expression is markedly enhanced in CHC patients with mild fibrosis [122]. *In vitro*, in Huh7 cells the co-overexpression of CYP2E1 and HCV core results in much stronger ROS production than in the cells overexpressing only one of these proteins [123]. Furthermore, we have shown that induction of CYP2E1 expression by HCV core and NS5A proteins results in a significant elevation of the levels of ROS [95, 113].

Additional input into ROS production during HCV infection can come from the unfolded protein response induced by the virus. In biopsies from CHC patients the areas which exhibited strong induction of oxidative stress also demonstrated signs of a pronounced UPR [124]. As discussed above, several HCV proteins activate UPR, resulting in an increased expression of the components of the protein-folding machinery. One of them is Ero1α, an enzyme that produces H_2_O_2_ [125, 126]. We have revealed that HCV core, but not NS5A, induces Ero1α expression, whereas the down-regulation of Ero1α expression using siRNAs leads to a decrease in hydrogen peroxide levels [95, 113]. Current concepts in the redox field claim that all peroxide in the ER lumen is effectively scavenged by peroxiredoxin 4 and glutathione peroxidases 7 and 8 [127, 128], and the effects of Ero1α reflect a dysregulation of calcium signaling. However, the ROS decrease due to Ero1α knock down may also reflect a direct suppression of H_2_O_2_ production by this enzyme. Certain mutations in Ero1α were shown to influence H_2_O_2_ production, ER oxidation, and cell toxicity [129], allowing to speculate that there may be other factors, like protein-protein interactions capable of blocking the process of Ero1α-directed H_2_O_2_ production/detoxification, which may be triggered by HCV proteins.

Different genotypes of HCV exhibit different abilities to induce oxidative stress [130]. Pro-oxidant activity decreases in the following sequence: 1a/b>4>2a/c>2b>3a. Considering the strong input of viral proteins into the oxidative stress *per se*, it is tempting to interpret this sequence as a reflection of the difference in (total) ROS induction in response to the combined activities of individual genotype-specific viral proteins.

### HCV and antioxidant defense pathways

HCV infection induces oxidative stress and simultaneously activates the antioxidant defense system. The oxidative stress response system is comprised of various enzymes capable of direct scavenging of ROS (phase II enzymes) and of the enzymes that are responsible for the biosynthesis and recycling of oxidized glutathione and other antioxidants. A considerable number of genes encoding these enzymes are controlled by the transcription factor Nrf2 [27]. Multiple evidence demonstrates that HCV strongly modifies the status and regulation along the Nrf2/ARE axis. A study by Carvajal-Yepes *et al.* reported suppression of this pathway by the full-length HCV or by a combination of the nonstructural proteins together with HCV core [131]. This block of the defense pathway was due tNS3-mediated sequestration of small Maf protein, which normally forms a heterodimer with Nrf2, and thus retains the latter outside the nucleus. However, other groups presented evidence for activation of the Nrf2/ARE pathway using the productively replicating *in vitro* HCVcc system, and CHC patient liver biopsies [132, 133]. Our group has shown that this activation is exerted by five viral proteins: core, NS5A, NS4B, E1, and E2 [74]. Amongst these three latter reports which observed an activation of the Nrf2/ARE axis by HCV, the only discrepancies relate to the protein kinase(s) responsible for the activation of Nrf2/ARE pathway: Burdette *et al*. assigned Nrf2 phosphorylation to mitogen-activated protein kinases [132], whereas our study showed that Nrf2 is phosphorylated by protein kinase C (PKC) in response to ROS, and by casein kinase 2 and phosphoinositide-3-kinase (PI3K) in a ROS-independent manner [74]. Jiang *et al.* specified that Nrf2/ARE activation was promoted by an inhibitory phosphorylation of glycogen synthase kinase 3β (GSK3β) [133]

Discrepant data on the status of the Nrf2/ARE pathway in HCV infection cannot be resolved by a sole consideration of the levels of proteins encoded by the Nrf2-dependent genes. For example, data were presented on the elevated levels of heme oxygenase 1 (HO-1) both in the infected cells and in the livers of chronic HCV carriers [134–137]. Three other groups presented a contradictory set of data showing an efficient down-regulation of HO-1 expression in the liver biopsies of CHC patients, cells expressing HCV proteins or harboring its subgenomic replicon [138–142]. A large set of transcriptomic and proteomic data based on HCV infected cells and liver tissues has been published but still does not clarify the issue. On one hand, human hepatocytes overexpressing the HCV polypeptide exhibit higher levels of expression of several Nrf2-dependent genes such as microsomal glutathione-S-transferase 3 (MGST3) or metallothionein 1F [143]. We also observed the transcriptional and translational up-regulation of the expression of HO-1 and NAD(P)H:quinoneoxidoreductase 1 (Nqo1) in Huh7 cells overexpressing HCV core and NS5A proteins as well as their truncated variants [144]. Similarly, it has been shown that HCV infection leads to an enhanced expression of two classical Nrf2 target genes [27]: glutathione reductase (*in vitro*) and glutathione synthase (*in vivo*) [145]. On the other hand, for the acute infection in a cell culture system Blackam *et al.* as well as Walters *et al.* reported a down-regulation of a wide spectrum of antioxidant defense proteins such as catalase, Nqo1, and glutathione-metabolizing enzymes [146, 147].

The exact mechanisms underlying these discrepancies remain to be elucidated, but it is tempting to speculate that the activation status of the Nrf2/ARE pathway during HCV infection may depend on the level of active TGF-β1 in the particular patient or system. Indeed, recent data have shown that exogenous TGF-β1 prevents activation of Nrf2 [133]. Chronic hepatitis C is accompanied by elevation of the levels of this cytokine in blood (serum) of the patients [148, 149]. The highest TGF-β1 levels are detected in patients with cirrhosis [150]. At the same time, standard therapy with interferon and ribavirin, as well as treatment with anti-fibrotic drugs, lead to a significant decrease in the TGF-β1 levels [151, 152]. Given the interdependence of the activation status of Nrf2/ARE pathway on the level of active TGF-β1, the huge variability of TGF-β1 levels in the patient cohorts involved in the above-mentioned studies may explain the contradictory observations regarding Nrf2/ARE-signaling. Another more mechanistic explanation is that the level of oxidative stress is bi-phasic: at low/moderate concentrations, ROS act as second messengers and trigger intracellular signaling cascades to switch on the antioxidant defense. When the level of ROS exceeds the capacity of the defense mechanisms, ROS start to irreversibly damage biomolecules and consequently inhibit the expression of antioxidant genes, activity of the phase II enzymes, *etc.*, thus ultimately triggering cell death. Differential Nrf2/ARE-signaling would then result from differences in the intensity and duration of oxidative stress in the described cohorts.

The antioxidant defense system also comprises glutathione peroxidases, peroxiredoxins, superoxide dismutases, catalase and other proteins. The nonstructural proteins of HCV down-regulate expression of SOD1 and SOD2 and induce catalase, whereas HCV core enhances the expression of SOD2 [153]. Increased levels of *sod2* mRNA are also observed in the HCVcc system [145], although no significant changes were reported in the catalase or dismutases levels in CHC patient livers [138]. HCV infection also increases the expression levels of GPx1 and GPx4 [145], implicated in protection against lipid peroxides. In addition, HCV NS3/4A protease was recently shown to cleave GPx8 [154]. This enzyme scavenges hydrogen peroxide produced in the ER by Ero1α (see above) [95, 128]). Interestingly, NS3/4A cleaves a small cytoplasmic tip of GPx8 molecule, however, this appears not to affect its enzymatic activity [154].

### Roles of ROS in the HCV life cycle

Reactive oxygen species influence various stages of the virus life cycle. Exogenous hydrogen peroxide inhibits HCV replication, and this effect is calcium dependent [155, 156]. The exact mechanisms of this effect remain unknown but it is already clear that they are not related to the stability of HCV genomic RNA. A similar calcium-mediated effect was recently observed for endogenous production of ROS as a result of the ER overload response in the HCV-infected cells [157]. Knock-down of GPx1 and GPx8, as well as of SOD1 or SOD2, in the infected cells had no significant impact on HCV replication [145, 154]. Another effect that ROS appear to have on HCV is an increase in HCV genome heterogeneity, which may contribute to the evolutional survival of the virus by ensuring viral escape from the immune system (during establishment of infection as well as during treatment) [158, 159]. Indeed, patients with a null genotype of glutathione-S-transferases, namely *gstt1* and *gstm1*, are characterized by decreased rates of the spontaneous resolution of acute HCV infection [160].

There may possibly be a direct link between the HCV core-induced oxidative stress and virion production. Formation and release of viral particles depend on the accumulation of lipid droplet (LDs). Core protein is known to localize to the surface of LDs and to induce morphological changes to LDs. HCV is also known to alter cell metabolism towards increased lipid biosynthesis and trafficking, decreased lipolysis and stabilization of LDs [161]. Attachment of HCV core to a phospholipid layer template depends on the presence of the hydrophobic domain at aa 117-169, which directs the formation/reconstitution of the nucleocapsid particles (while HCV core aa 1-117 is soluble and monodispersed) [162]. Studies of the C-terminally truncated HCV core variants demonstrated that variants lacking 39 to 42 C-terminal amino acids can neither associate to lipids/lipoproteins/LDs, nor form multimers, and cannot be secreted into the cell culture medium [162–164]. At the same time, structural studies in cells expressing core variant-GFP chimeras demonstrated that the core domain dubbed D2, when fused to GFP, is sufficient to induce the accumulation of large LDs containing the chimeric core proteins [165]. It is hardly surprising that the productive infection by HCV, *i.e.* the release of viral particles, is exquisitely predetermined by the presence of the D2 core domain [166]. At the same time, HCV core C-terminus carries the motive responsible for the protein association with mitochondria. Interaction of this domain of HCV core with mitochondria increases Ca^2+^ entry and subsequently elevates generation of ROS by mitochondrial electron transport complex I [167]. These two motives/domains appear to overlap; a sub-domain of D2 at aa 144-165 is involved in both the formation of LDs and mitochondrial association. In this context, it is important to mention that both the disruption of mitochondria and oxidative stress significantly augment lipogenesis and LD formation [168, 169]. Thus, one and the same domain of HCV core appears to mediate the induction of oxidative stress *via* binding to mitochondria, and the stimulation of lipid biosynthesis *via* enhancement of oxidative stress. The accumulation of lipids into the lipid droplets is vitally important, as it favors HCV assembly. Thus, HCV appears to exploit oxidative stress and mitochondrial injury to ensure its own propagation.

At the same time, an excessive oxidative stress may hamper viral viability. Down-regulation of GPx4 [145], an enzyme that offers protection from the accumulation of lipid peroxides, induces a moderate suppression of HCV replication and strong suppression of virion infectivity [170]. Similarly, the down-regulation of GPx8 by RNA interference impairs virus particle assembly [154]. Moreover, a SEC14L2 protein, also known as tocopherol-binding protein 1, was recently identified as the cellular factor crucial for pan-genotype HCV replication in the hepatocytes [171]. Its effect was attributed to enhanced uptake and activity of vitamin E [171], one of two *bona fide* antioxidants [26] that, like GPx4, protect lipids from the peroxidation. Saeed *et al.* showed that SEC14L2 ensured protection of HCV replication from lipid peroxidation, since the effect of the protein was observed even in cells harboring subgenomic replicons of the virus [171]. In our previous review we hypothesized that oxidative stress leads to the accumulation of lipid peroxides that might be incorporated into virus particles and prevent the effective binding of “oxidized” virions to low density lipoprotein receptors, which would hamper the initial state of HCV entry [17]. Indeed, we have lately shown that HCV virus particles produced from cells with suppressed GPx4 levels have lower infectivity and fusion activity, and that both effects could be prevented by tocopherol [145]. Thus, lipid peroxides inhibit two steps of the HCV life cycle: replication and production of infective virions.

Reactive oxygen species may also promote the translation of the HCV genome [172, 173]. This effect is mediated through the induction of UPR, particularly the activation of its PERK branch [172]. PERK phosphorylates eukaryotic initiation factor 2α (eIF2α), leading to the suppression of cap-dependent translation and often switching ribosomes to cap-independent translation, which might account for the observed effects [108, 174]. However, contrary observations have also been reported [156].

Finally, ROS may suppress HCV replication through modulation of the Nrf2/ARE pathway. A recent study of Yu *et al.* revealed that induction a classical Nrf2-dependent gene, HO-1, down-regulated HCV replication through induction of interferon α and inhibition of NS3 protease activity by bilirubin, HO-1 product [142].

### Roles of ROS in HCV-associated pathology

#### Markers of oxidative stress during chronic hepatitis C correlate with liver damage

Hepatitis C virus-induced oxidative stress contributes to the development of various virus-associated pathologies. This is supported by correlations between the stress markers and the incidence and/or severity of HCV-related (co)morbidities. Levels of oxygen radicals, as quantified by EPR, correlate with histological disease activity [45]. For example, serum MDA or urine 8-isoprostane levels correlate with the stage of fibrosis [53, 54, 65]. Various oxidative stress markers positively correlate with the levels of alanine aminotransferase (ALT) and aspartate aminotransferase (AST), indicative of liver damage [47]. Moreover, an increase in these markers in patients with persistently normal aminotransferases predicts a subsequent increase in ALT and AST activities. Positive correlations were revealed between the dROM index (*i.e.* total prooxidant activity) of the sera and the fibrosis score [71]. Oxidative DNA modifications also serve as markers for liver necroinflammatory activity and tissue damage [46, 53, 54, 63]. Accordingly, an inverse correlation exists between the liver damage and the extent of intrahepatic Nrf2 activation [133]. Recently a study of Huang *et al.* showed that a co-infection with HIV enhances liver abnormalities visualized by ultrasonic study [59]. Interestingly, in these co-infected patients ALS/AST levels correlated with such marker of oxidative stress as the level of GSSG.

#### Production of proinflammatory cytokines during HCV infection is triggered by ROS

**Figure 2:**
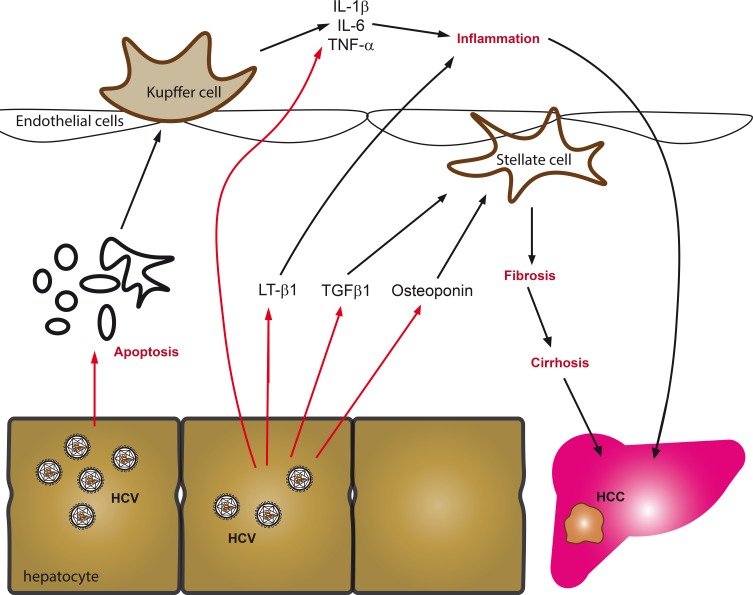
Key mechanisms driving fibrosis in chronic hepatitis C The predominant sources of proinflammatory cytokines and chemokines in chronic viral hepatitis are generally infected hepatocytes, activated Kupffer cells or circulating leukocytes. These cells are stimulated by hepatitis viruses to produce cytokines in response to activation of cellular signaling cascades, virus-induced oxidative stress, apoptosis of infected cells or direct activation of innate and adaptive immunity. Amongst the predominant cytokines that are produced are tumor necrosis factor α (TNF-α), interleukines 1β (IL-1β), 6 (IL-6), 8 (IL-8), as well as lymphotoxin (LT). In addition to stimulation of inflammation, infected hepatocytes release several fibrogenic mediators including besides ROS, the phosphoprotein osteoponin and TGF-β1, all of which activate hepatic stellate cells or myofibroblasts, which in turn amplify cytokine secretion and start to produce and deposit extracellular matrix components.

Chronic hepatitis C is characterized by persistent liver inflammation [34]. This is manifested by the enhanced production of proinflammatory cytokines and chemokines, including tumor necrosis factor α (TNF-α), interleukins 1β (IL-1β), 6 (IL-6), 8 (IL-8), as well as lymphotoxin (LT) (Figure 2). Their production is attributed to liver Kuppfer cells and to circulating leukocytes [175], triggered by the apoptotic death of infected hepatocytes [176]. HCV can also facilitate IL-1b production in macrophages through production of ROS by NADPH oxidases [177]. Production of these cytokines can also occur in infected hepatocytes. Indeed, HCV-infected Huh7.5 cells demonstrated markedly enhanced levels of LT-α and -β, TNF-α and IL-8 [178]. The expression of TNF-α and IL-8 can be directly attributed to the effect of ROS, as their genes are controlled by the ROS-sensitive NF-κB pathway [179]. This is in line with observations that show activation of NF-κB and STAT3 by HCV core, NS4B and NS5A proteins through enhanced ROS production and altered Ca^2+^ homeostasis [84, 157, 180]. Accordingly, levels of the antioxidants in the liver are higher in patients with mild compared to moderate-to-severe inflammation and fibrosis [56].

#### ROS promote liver fibrosis

Liver fibrosis is another pathology strongly associated with chronic hepatitis C. Fibrosis represents an excessive production of the extracellular matrix (proteoglycans, collagen, *etc.*) by myofibroblasts [181]. Its mechanisms have been intensively studied in many laboratories, however, the origins of the extracellular matrix-overproducing myofibroblasts in different diseases are still disputed. It has been proposed that myofibroblasts can originate from the hepatic stellate cells (HSCs), portal fibroblasts, or even hepatocytes during epithelial-mesenchymal transition. Although conversion of hepatocytes into myofibroblasts was shown *in vitro* by several groups (for example, [182]), such event is unlikely to occur *in vivo* [183]. Data supporting the conversion of hepatocytes into myofibroblasts are scarce, even in animal models of fibrogenesis [184]. The current concepts imply that myofibroblasts in CHC originate from activation of HSCs [181].

The processes of HSC activation and proliferation are triggered by several cytokines: transforming growth factor β1 (TGF-β1), platelet-derived growth factor (PDGF) and connective tissue growth factor (CTGF) [185, 186] (Figure 2). In CHC, levels of TGF-β1 are elevated in serum and liver, and correlate with the fibrosis score [187, 188]. TGF-β1 is produced by several types of cell, including Kupffer cells and hepatocytes [189]. Its expression/secretion in the infected hepatocytes is promoted by core, NS3/4A, NS4B, and NS5A proteins through ROS- and calcium-dependent mechanisms [178]. Furthermore, the processing of TGF-β1 polypeptide depends on the uptake of calcium ions by mitochondria, and is thus ROS-dependent [178]. In a hepatocyte-HSC co-culture model, ROS-dependent increase in TGF-β1 production triggers activation of stellate cells and production of extracellular matrix.

Another mediator of fibrogenesis in CHC is osteoponin, a phosphoprotein, the level of which gradually increases with the progression of fibrosis [190–192]. As in the case of TGF-β1, the level of osteoponin in the HCV-infected hepatocytes increases with the increased levels of ROS production and altered calcium homeostasis [193]. Besides activating HSCs, osteoponin suppresses the expression of matrix metalloproteinase 13, shutting down the removal of extracellular matrix and forbidding fibrosis resolution [194].

Activation of the hepatic stellate cells can be triggered not only by cytokines, but also by the uptake of apoptotic bodies from HCV-infected hepatocytes [195]. HSCs activation is also promoted by the extracellular HCV core protein [196] as well as by the expression of E1 glycoprotein on/in the stellate cells [81], although the physiological relevance of these mechanisms remains obscure. Production of ROS in hepatocytes via CYP2E1 is essential for the activation and proliferation of HSCs [197]. Both HCV core and NS5A proteins induce CYP2E1 [95, 113], indicating that this mechanism may also contribute to HCV-induced fibrogenesis.

Hepatocellular carcinoma.** HCV-induced oxidative stress contributes to the development of hepatocellular carcinoma. Moreover, levels of oxidative stress markers in CHC patients correlate positively with the probability of development of HCC [198] and can serve as prognostic markers for HCC recurrence in chronic hepatitis C patients who underwent liver transplantation [199]. Carcinogenesis is orchestrated by multiple ROS-mediated processes. Firstly, the increased ROS production, in particular in the nucleus, leads to DNA damage, accumulation of mutations and to genetic instability [200]. Elevated levels of such oxidative stress markers as 8-oxoG or thioredoxin are often found in CHC patients who develop liver cancer [201–205]. Moreover, levels of 8-oxoG increase with the progression of liver disease. Furthermore, patients with advanced stages of disease exhibit shortening of telomeres and elevated levels of telomerase due to the methylation of its promoter [205]. HCV can also suppress excision of oxidized nucleobases from DNA by down-regulation of expression of Nei (endonuclease VIII)-like protein 1 (NAIL1) [79], an enzyme that catalyzes this reaction [206]. The virus thus blocks the damage response to DNA lesions [206], though no changes were reported for the expression of other glycosylases performing base excision and reparation, such as Nei (endonuclease VIII)-like protein 2 (NAIL2) or oxoguanine DNA glycosylase (OGG1) [79].

**Figure 3:**
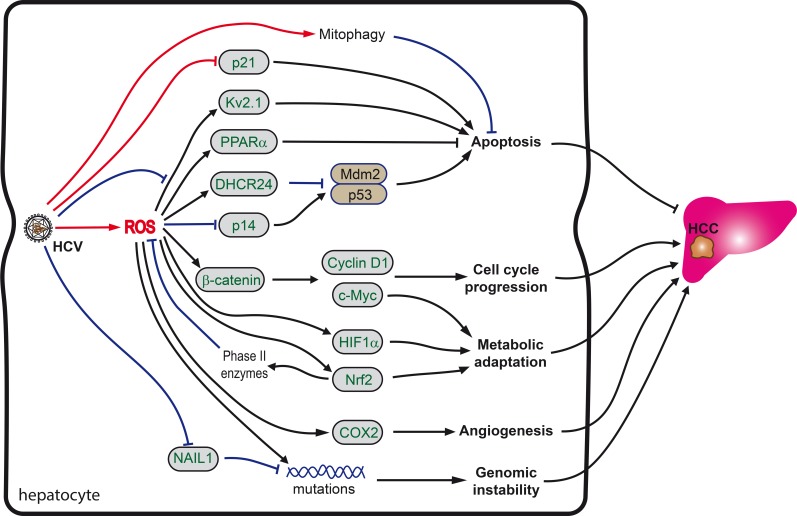
Role of oxidative stress in HCV-induced hepatocarcinogenesis Carcinogenesis is orchestrated by a combination of HCV as well as ROS-mediated processes. Increased ROS levels directly cause increased genetic mutations. However, if ROS levels as well as other stress signals are not high enough to induce apoptosis, pro-survival and repair programs, including the Nrf2/ARE pathway are activated, which in turn overcome cell cycle arrest, induce mitophagy and reprogram metabolism. See text for more details.

Secondly, in some cells the oxidative stress, instead of inducing apoptosis, switches on the pro-survival programs, including the Nrf2/ARE pathway, which overcome cell cycle arrest, mitophagy and metabolic reprogramming (Figure 3). Activation of Nrf2 transcription factor basically leads to the partial scavenging of ROS, thus preventing their accumulation to lethal doses [132]. Cell cycle progression during enhanced ROS production is achieved through activation of β-catenin, leading to enhanced expression of c-Myc transcription factor as well as of cyclin D1 and other molecules [70]. Increased ROS production also leads to the up-regulation of expression of 24-dehydrocholesterol reductase (DHCR24), disrupting binding of p53 to Mdm2, thus preventing apoptosis [207]. HCV also counteracts apoptosis through the activation of peroxisome proliferator-activated protein α (PPARα) [208] and through suppression by NS5A protein of the potassium ion channel Kv2.1 [209]. It is noteworthy that both events involve reactive oxygen species as mediators. In addition, ROS suppress the expression of p14 protein that is implicated in induction of the proapoptotic p53-Mdm2 pathway [210]. Additional input into the survival of HCV-infected cells under chronic mitochondrial dysfunction comes from the induction of mitophagy, an event of autophagosomal removal of damaged mitochondria [211].

Thirdly, HCV/HCV proteins interfere with the activity of the cyclin-dependent kinase inhibitor p21/Cip1/WAF1, a crucial protein in the regulation of cell cycle arrest, DNA replication and repair, cell differentiation, senescence, and apoptosis [212–214]. It is up-regulated in response to oxidative stress, promoting cell survival [214]. The p53-p21 axis appears to delegate the pro-survival duties to the Keap1-Nrf2 stress pathway [215, 216]. The level of Nrf2 was shown to be upregulated through a direct binding of Nrf2 to p21 [215, 216]. P21 competes with Keap1 for Nrf2 binding, thus inhibiting Keap1-dependent Nrf2 ubiquitination, resulting in the stabilization of Nrf2 protein. Nrf2 is essential for the antioxidant effect of p21: the ectopic expression of p21 enhances cell survival in response to H_2_O_2_ in MEF-Nrf2^+/+^ but not in MEF-Nrf2^−/−^ cells [217]. The p21-mediated activation of the Nrf2 signaling pathway was suggested to be the initial defense mechanism for reducing ROS to protect cells from oxidative damage under low stress conditions [214, 218]. At a moderate level of oxidative stress involving DNA damage, p21-mediated cell cycle arrest would be activated to allow time for DNA repair. At high levels of oxidative stress, as the point of no return, p21-mediated apoptosis would be induced. The latter requires accumulation of ROS, and is induced by the CDK-binding domain of p21/Waf1 [219] and the suppression of the Nrf2 antioxidant response pathway, detectable as a decrease in Nrf2 protein levels at the high levels of oxidative stress [217]. However, HCV/HCV proteins interfere with the p21 activities and subsequent regulation of cell cycle activity. Specifically, HCV core forms a complex with p21/Waf1 protein and induces a marked post-transcriptional reduction of p21/Waf1 expression [220, 221]. Interestingly, HCV core-induced inhibition of the expression of p21/Waf1 is abrogated by deletion of the C-terminus of HCV core [92], which is also involved in NF-κB-dependent apoptotic cell death [222] and induction of the expression of CYP2E1 [95]. NS5A is also implicated in the down-regulation of the p21/Waf1 promoter activity [223]. Both effects of HCV proteins on the expression of p21/Waf1 make the Nrf2-depedent stress response less sensitive to p21-regulation. According to Villeneuve *et al.,* this would interfere with cell survival under low to moderate stress, but ensure continuous cell proliferation under severe stress [217]. Indeed, in mice, depletion of p21 leads to a continuous proliferation of severely injured hepatocytes and rapid tumor development [91]. In lines with this, p21/Waf1 expression levels in the cancerous tissues appear to be significantly reduced compared to those in the noncancerous tissues [224, 225]. Furthermore, the histological negativity for p21/Waf1 serves as a negative prognostic factor for the survival of patients with HCC after resection [226], although a contrary finding has also been reported [227].

Fourthly, carcinogenesis in chronic hepatitis C is associated with long-lasting inflammation, specifically associated with lymphotoxin (LT) signaling [228, 229]. LT production is not triggered by ROS: it is induced by HCV RNA-dependent RNA polymerase [229] that does not affect cellular redox status [74]. However, site-specific oxidation of methionine residues impairs the bioactivity of LT and prevents its binding to the TNF receptors 1 and 2 [230]. The latter leads to dysregulation of ligand-induced signaling with respect to the activation of “protective” proteins such as SOD2 against “killing” proteins such as proteases [231], eventually resulting in the aberrant induction of apoptosis. However, one should keep in mind that liver tumors can develop even in the absence of signs of hepatic inflammation, as was exemplified by the experiments with HCV core-transgenic mice [208, 232].

Last but not the least, despite a lack of systematic evaluation of HCV's influence on the carbohydrate metabolism, evidence is accumulating of a metabolic adaptation of chronically infected cells to oxidative stress, which may appear to be critical for the survival of the transformed cells. In support of this, HCV-mediated oxidative stress leads to the induction of the hypoxia-inducible factor 1α (HIF1α) [233]. HIF1α, together with Nrf2 and c-Myc, controls expression of key enzymes of glycolysis, glutaminolysis and gluconeogenesis [234] and is known to induce a Warburg effect and glutamine dependence, typical of the majority of tumors [234–238].

Besides triggering tumorigenesis, oxidative stress supports the survival and propagation of existing tumors by promoting angiogenesis. The latter could be achieved *via* ROS-dependent up-regulation of the expression of cyclooxygenase 2 (COX2). It was shown long ago that COX2 overexpression enhances the production of prostaglandin E2 and induces the formation of new vessels [239, 240].

### HEPATITIS B VIRUS

#### Hepatitis B biology

HBV is the leading cause of hepatocellular carcinoma; about 350 million people worldwide are chronic carriers. Of those who are infected around the time of birth, 90% develop chronic as against less than 10% of those infected after the age of five [241]. Chronic infection is usually asymptomatic but cirrhosis and liver cancer may eventually develop [242]. These complications result in the death of 15 to 25% of those with chronic disease [243]. HBV is an enveloped DNA virus with an icosahedral nucleocapsid core structure. The virus is small, diameter 42 nM, and its tropism is confined to hepatocytes. HBV is classified as the prototype member of the Hepadnaviridae. The genome consists of circular, only partially double-stranded DNA. One end of the full length strand is linked to the viral DNA polymerase. Viral replication includes RNA intermediates. Therefore, viral genomic DNA has to be transferred to the cell nucleus, where the partially double-stranded viral DNA is then made fully double-stranded by viral polymerase and transformed into covalently closed circular DNA (cccDNA). This cccDNA serves as a template for transcription and translation of the four viral proteins C (core, HBcAg), X (HBx), P (DNA polymerase), and S (surface antigen, HBsAg). The function of the protein coded for by gene X is not fully understood but is associated with the development of liver cancer. It stimulates genes that promote cell growth and inactivates growth regulating molecules. In chronic HBV infection, the host immune response causes both hepatocellular damage and viral clearance. In particular, the adaptive immune response, such as virus-specific cytotoxic T lymphocytes (CTLs), contributes to most of the liver injury by eliminating infected hepatocytes and stimulating production of inflammatory cytokines [244]. Although liver damage is initiated and promoted by the CTLs, direct interaction of HBV within the hepatocytes is also thought to have a detrimental effect on liver physiology.

#### Oxidative stress during hepatitis B

Patients with chronic hepatitis B exhibit signs of pronounced oxidative stress. Levels of oxygen radicals in liver specimens from these patients exceed the levels in healthy people by 1.5-4 orders of magnitude [44, 45]. Immunohistochemical analysis from such liver biopsy specimens also reveals elevated levels of DNA oxidation products such as 8-oxoG [46, 245] as well as lipid peroxidation products [246].

Patients with hepatitis B exhibit signs of oxidative stress not only in the liver but also in plasma/sera. Chronic hepatitis B is accompanied by an increase in total oxidant status and a concomitant reduction of total antioxidant status [247, 248]. Plasma/serum of these patients was also characterized by the elevated levels of ROS, including H_2_O_2_ [248, 249], and oxidation products of lipids [247, 250–253], and proteins [253, 254]. Moreover, some of these products, such as 8-oxoG, were elevated even in urine, indicating a possibility to use them as biomarkers of chronic liver disease [255]. HBV infection also leads to a decreased concentration of total glutathione (GSH), an elevated level of its oxidized form (GSSG) and an abnormal GSSG/GSH ratio in plasma and blood cells [73, 245, 250, 251, 253].

Oxidative stress is not just a hallmark of chronic HBV infection and advanced liver disease; it is also observed in acute and occult hepatitis B, as well as in asymptomatic HBV infections. Occult hepatitis B infection is characterized by increased levels of ROS in lymphocytes and consequent DNA damage [249]. Duygu *et al.* reported a decreased total antioxidant status and changes in several oxidative stress markers (*i.e.* free SH groups) even in patients with a non-symptomatic course of infection [247] (though other studies refute these findings [248]). The increase in the levels of the lipid oxidation products and the reduction in levels of reduced glutathione are even more pronounced during acute as compared to chronic HBV infection [73, 250]. However, the most dramatic changes have been described in hepatitis B patients with liver cirrhosis [248] and with acute-on-chronic hepatitis B liver failure (ACHBLF) [256–258].

#### HBV enhances ROS production through Ca^2+^-mediated mitochondrial dysfunction and unfolded protein response

Studies of the mechanisms by which HBV induces oxidative stress are much less advanced than those concerning HCV. The mechanisms have mainly been studied in infected cells, in cell lines producing viral proteins and in laboratory animals. These studies have shown that HBV-mediated ROS production is triggered by three viral proteins, HBx [69, 259–262], HBsAg [263, 264], and HBcAg [265] antigens.

**Figure 4:**
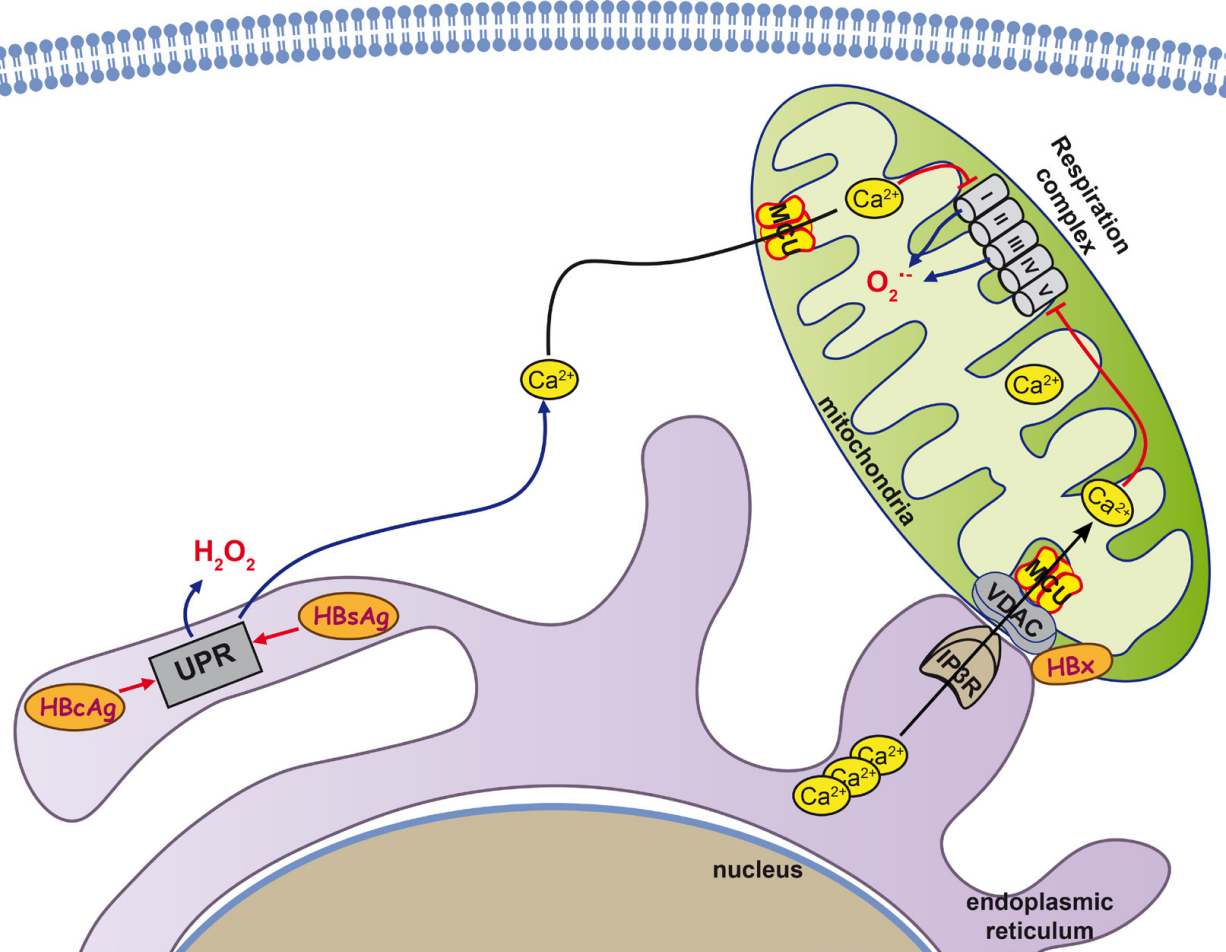
Mitochondria as key organelle in HBV-induced hepatocarcinogenesis Three HBV proteins, HBx and HBs and HBc antigens are known to interact with mitochondria and contribute to the induction of oxidative stress. HBx interacts with voltage-dependent anion channel 3 (VDAC3) and cytochrome c oxidase subunit III (COXIII), a subunit of the cytochrome c oxidase respiratory complex IV, and thus increases Ca2+ levels in both cytoplasm and mitochondria, leading to permeability transition pore (PTP) dysregulation. In addition, in infected cells HBsAg and HBcAg variants tend to accumulate in the ER and induce ER stress as well as an unfolded protein response (UPR). The UPR triggers release of the Ca2+ into the cytoplasm, with subsequent enhancement of ROS production.

HBx-expressing cells exhibit a reduced enzymatic activity of the respiratory complexes I, III, IV, and V, and a decreased expression of several of their subunits [259]. Their dysregulation is thought to cause a loss of mitochondrial membrane potential and an enhanced production of ROS. Although HBx protein localizes to different compartments: cytoplasm, nucleus, and mitochondria (mt) [266–268], the induction of oxidative stress is thought to be mainly due to its mt localization and its effect(s) on this organelle (Figure 4). In mitochondria, HBx was shown to be bound to the outer membrane [268]. Several regions were reported to mediate mitochondrial localization: amino acid residues 68-117 [266, 269], 111-117 [270], and 121-154 [261]. Their deletion results in the abrogation of pro-oxidant activity, reversal of depolarization of the mitochondrial membrane, and loss of the ROS-dependent effects in the expressing cells [261, 269, 271, 272]. The decisive role of the HBx-mt association in the induction of oxidative stress is supported by the finding of elevated mt levels of superoxide anion and DNA oxidation products (*i.e.* of 8-oxoG) [261].

Two key mitochondrial proteins have been identified as the HBx-binding partners: voltage-dependent anion channel 3 (VDAC3) [271] and cytochrome c oxidase subunit III (COXIII) [273, 274]. VDAC3 forming an ion channel localized on the outer mt membrane is implicated in the formation of the mt permeability transition pore (PTP), dysregulation of which is one of the best-known mechanisms of oxidative stress induction [88]. VDAC and PTP are specifically involved in control of the transport of calcium ions through the outer membrane of mitochondria. HBx protein was shown by M. Bouchard's group to cause a pronounced increase in Ca^2+^ levels in both cytoplasm and mitochondria, leading to the profound PTP dysregulation [268, 275, 276]. No similar study was done for VDAC3 protein but HBV-unrelated studies indicate that accumulation of calcium ions in mitochondria in response to histamine are enhanced by the overexpression of VDAC3 and suppressed by its down-regulation by RNA interference [277]. Hence, any effect HBx might have on VDAC3 would inevitably lead to modulation of the mt content of calcium ions and the induction of oxidative stress. The effect(s) of HBx on VDAC1 or VDAC2 has not been assessed yet.

The second mitochondrial partner protein of HBx is COXIII [273, 274, 278], which is one of the subunits of the cytochrome c oxidase respiratory complex IV [279]. HBx causes an increased expression of COXIII and stimulates complex IV activity [273, 274]. A direct interaction between HBx and COXIII was identified in a yeast two-hybrid system and by immunoco-precipitation [280]. However, as HBx localized to the outer mt membrane [268], whereas COXIII is localized at the inner membrane [279], these data need to be validated. In view of this, it is possible that HBx does not directly bind to COXIII but interacts indirectly *via* some yet unidentified protein(s).

Another protein of hepatitis B virus responsible for the induction of oxidative stress in infected cells is HBsAg [281]. It is encoded by ORF, which supports the formation of three variants of surface antigen: small (S-domain), middle (preS2-S domains), and large (preS1-preS2-S domains) [282]. HBsAg is excreted from the cell even in the absence of other components of the virus [283]. However, some naturally-occurring mutants of the small HBsAg exhibit a reduced ability to be secreted and instead accumulate in the ER [264]. This was also reported for the natural variants of the large antigen with short truncation(s) in preS1 and/or preS2 domains [281]. The accumulation of the HBsAg variants in the ER results in ER stress and a consequent UPR [264, 281]. In turn, HBsAg-induced UPR induces a release of calcium ions into the cytoplasm, with subsequent enhancement of ROS production. An additional source of oxidative stress is a reduction of expression of the antioxidant defense Nrf2/ARE pathway [283] and protective enzymes, such as catalase and HO-1, in particular [264]. However, a study on the cohort of chronic hepatitis B patients failed to find any association between the mutations in pre-S domains of HBsAgs and the level of oxidative stress (as manifested by the DNA damage) [284].

ER retention of the truncated middle HBsAg was also shown to trigger ROS production [285]. However, since the authors acknowledged the concomitant activation of the transcription factors NF-kB and AP-1 [285], one can suspect that the retention of the middle HBsAg induces not an UPR, but rather the ER overload response [286].

The third HBV protein that triggers ROS production is the HBcAg. The mechanism of its action is similar to that of HBsAg; naturally occurring mutants tend to accumulate in the ER, leading to a calcium efflux and concomitant oxidative stress [265].

#### Influence of HBV on antioxidant defense system

Hepatitis B virus strongly induces the Nrf2/ARE pathway of antioxidant defense [283, 287]. It has been observed both in infected cell cultures and in liver tissues of chronic hepatitis B carriers [283, 287]. These findings are consistent with several independent *in vitro* studies that show increased expression levels of classical Nrf2-dependent phase II enzymes, such as glutathione synthetase (GSS), catalytic (GCLC) and regulatory (GCLM) subunits of glutamate-cysteine ligase, and glutathione reductase (GR) [288, 289]. Interestingly, different genotypes of the virus stimulate the Nrf2/ARE pathway in different ways: genotype A activates the pathway to a much higher degree than genotype G [283]. In addition, HBV-infected cells demonstrated an enhanced expression of metallothioneins [289]. Transcription of these genes is ARE-dependent and thus exclusively controlled by nuclear factor erythroid 2 related factor 1 (NF-E2-related factor 1, or Nrf1) [290]. Activation of the Nrf2/ARE pathway in the infected cells is achieved by HBx and LHBs proteins of the virus [287]. In the case of HBx, the underlying mechanisms is ROS-independent and rather implies sequestering of the Nrf2 partner protein Keap1 *via* formation of a triple HBx-p62-Keap1 complex [287, 291].

However, there is a great deal of conflicting data concerning the status of the Nrf2/ARE pathway in HBV-infected cells and in HBV patients. For example, the induction of expression of Nqo1 in the study by Schaedler *et al.* [287] is challenged in two other studies, which report the suppression of Nqo1 due to HBx-mediated recruitment of DNMT3A methyltransferase to the Nqo1 gene promoter, thus leading to its hypermethylation [292, 293]. Other Nrf2-dependent genes, such as glutathione-S-transferases M3 (GSTM3) [256] and π (GSTP1) [294, 295], were also reported to be epigenetically suppressed in either HBV-infected or HBx-expressing cells. HBx can inhibit expression of phase II enzymes not only by epigenetic mechanisms, but also by interfering with regulatory elements/factors other than Nrf2/ARE. For example, HBx was shown to prevent the expression of genes encoding phase II enzymes in response to agents that activate C/EBP elements in their promoters [296]. Interestingly, for GSTP1, such inhibition occurs in the case of genotype D of the virus, but not genotypes A-C [294]. The suppression of these genes augments oxidative stress [297]. In support of this, sera of chronic hepatitis B patients display no activation of the Nrf2-dependent gene encoding thioredoxin, although this could be explained by the activation of this pathway/gene exclusively in the infected hepatocytes [62].

HBV also changes the levels of expression of other antioxidant defense enzymes that are not regulated by the Nrf2/ARE pathway. First, production of the virus leads to an increased expression of two omega 1 (GSTO1) [295] and kappa 1 (GSTK1) [298] isoforms of glutathione-S-transferases encoded by the Nrf2-independent genes [299]. Second, both HBV-producing transgenic mice and chronic hepatitis B carriers exhibit an increase in the liver of the expression of peroxiredoxin 1 [298]. Third, various experimental systems demonstrate that HBV induces SOD2 [289, 298, 300], although contradictory findings were also reported [301]. The discrepancies in these animal studies could be explained by different experimental set-ups: the induction was shown for 6-8 week old mice [300], whereas the suppression was described for old animals [301]. These aged mice also exhibited the markedly decreased levels of glutathione peroxidase, with no changes in catalase or SOD1 levels [301]; however, catalase might have been induced by the virus since HBV-infected patients demonstrate an increased intracellular activity of this enzyme [252].

HBV also suppresses the expression of proteins that are indirectly involved in the antioxidant defense system. Namely, HBx, was shown to inhibit the expression of selenoprotein P (SeP) [302] as well as of selenium-binding protein 2 (Selenbp2) [298]. SeP is a protein synthesized and excreted by liver cells [303, 304]. It's role is to bind to dietary selenium and to transport it to various organs. A decrease in SeP expression correlates with a reduction of selenium levels in the organism, which in turn hampers the activity of various antioxidant selenoproteins such as GPx, GST, and thioredoxin reductases.

#### ROS as regulators of the HBV life-cycle: almost nothing is known so far

Data on the influence of ROS on the HBV life-cycle are scarce. It is not yet known whether oxidative stress can affect the early stages of infection, and/or regulate the activity of HBV DNA polymerase and HBV replication.

Past findings have indicated that H_2_O_2_ increases intracellular concentrations of the viral DNA, but this may simply be related to the oxidative damage-related increase in the number of sites available for HBV DNA integration [305]. However recently it was reported that H_2_O_2_ enhances HBV replication, whereas N-acetylcystein or overexpression of sirtuin 3 that alleviate stress, suppress replication of the virus [262].

The effect of ROS on gene transcription is ambiguous. On the one hand, hydrogen peroxide at high concentrations (>0.1 mM) decreases the expression and secretion of HBsAg and HBeAg and consequently reduces the number of produced virions [306]. On the other hand, H_2_O_2_ (0.5-1 mM), as well as anticancer agents (*i.e.* doxicyclin, adriamycin/doxorubicin) which trigger its production in the cell, activate the expression of HBx by stabilizing its mRNA and protein [307, 308]. Min *et al*. also demonstrated that ethanol enhanced transcription from the core and pre-S1, but not from pre-S1/S and X promoters [309]. Such activation is achieved through induction of a ROS-producing CYP2E1 and activation of several hepatic transcription factors that bind to these promoters.

Secondly, hydrogen peroxide was shown to promote HBV capsid assembly [310]. This enhancement was revealed in several systems, including cell-free experiments involving Hsp90 protein, thus demonstrating that H_2_O_2_ can influence the conformation of the Hsp90/capsid complex.

Finally, oxidative stress enhances the integration of hepadna virus DNA into the host cell genome, as shown for human HBV and duck hepatitis B virus infection in the cells treated with H_2_O_2_at micromolar concentrations [305, 311]. In this context, the increase in the intracellular concentration of viral DNA relates to the oxidative damage-related increase in the number of sites available for HBV DNA integration. It is noteworthy that all the above-mentioned effects in the HBV-infected cells depend on ROS but are not related to the activation (or suppression) of the Nrf2/ARE pathway [287].

#### ROS as mediators of HVB pathogenesis: an obscure subject

**Figure 5:**
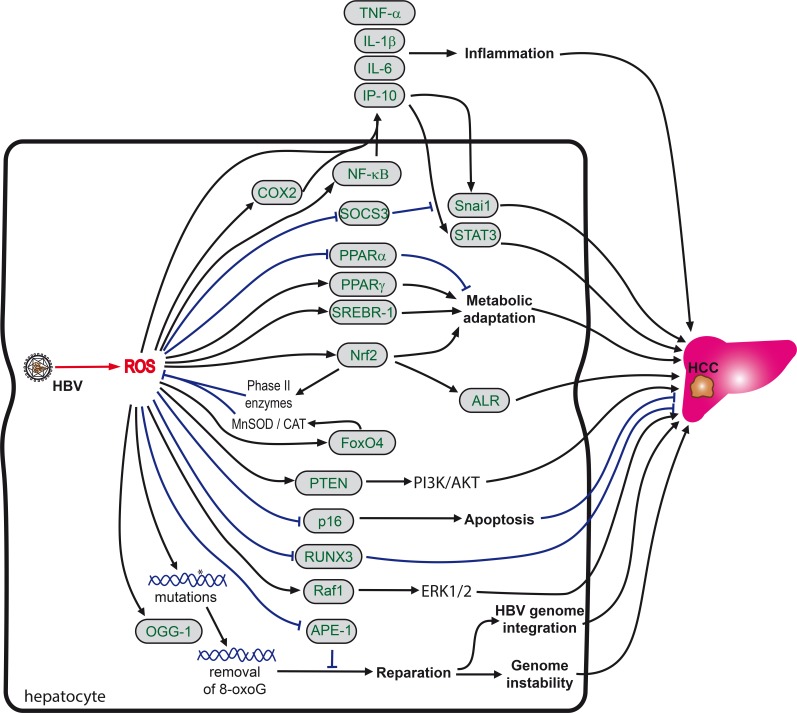
Role of oxidative stress in HBV-induced hepatocarcinogenesis HBV-induced oxidative stress promotes liver carcinogenesis via modulation of signaling cascades, resistance to ROS-triggered apoptosis, promotion of genome instability and metabolic adaptation of infected cells. So far analysis of the mechanisms by which HBV-derived ROS drive carcinogenesis are limited predominantly to *in vitro* systems based on HBx overexpression, however some observations have been made in biopsy materials. HBx activates proinflammatory NF-κB signaling via ROS generated in mitochondria. HBx induces, again in a ROS-dependent manner, the expression of cyclooxygenase 2 (COX2), which in turn amplifies proinflammatory cytokine signaling. Virally-induced ROS also activate the Nrf2/ARE pathway with subsequent induction of liver regeneration (ALR) protein, the forkhead transcription factor FOXO4 and alterations to the PI3K/AKT pathway. Additional metabolic alterations induced by HBx are due to induction of key transcription factors of lipid homeostasis, such sterol regulatory element binding protein 1 (SREBP-1), liver X receptor, C/EBP1 and peroxisome proliferator-activated receptor γ (PPARγ). HBx also induces oxidation of tumor suppressor phosphatase and tensin homolog protein (PTEN), which further enhances the activation of the PI3K/AKT pathway. In addition, in HCC tissues expression of OGG1, a DNA repair enzyme frequently is frequently (over)expressed and methylation of the promoter of a runt-related transcription factor 3 (RUNX3) gene is augmented. RUNX3 is a protein whose inactivation has been implicated in the development of various solid tumors. Finally, HBV also causes a ROS-dependent translocation of Raf-1 kinase to mitochondria.

HBV-induced oxidative stress is a crucial factor for establishment of chronic infection and the further development of liver inflammation and cancer (Figure 5). Patients with chronic hepatitis B have increased levels of sulfhydryl and lipid peroxidation [247] as well as hepatic oxidative DNA damage [245]. In HBV-infected children the level of oxidative stress markers correlates with the rate of chronicity of the disease [312]. The direct mechanisms underlying this effect are not known. However, Schaedler *et al.* reported that HBV-induced oxidative stress is accompanied by activation of the Nrf2/ARE pathway, leading to suppression of the immunoproteasome and probably to at least partial evasion from immune responses [287]. Interestingly, additional input into the suppression of antigen presentation in infected cells may be achieved *via* induction of proteasome-inhibiting factor CYP2E1 on the background of heavy alcohol consumption [313].

Oxidative stress also contributes to liver damage and inflammation. For example, an increase in MDA levels was observed in patients with an increased risk of an acute-on-chronic hepatitis B liver failure [256]. Levels of 8-oxoG in the liver determined by immunohistochemical tissue staining were shown to correlate with the activity grade of chronic hepatitis [46] and with the amount of aspartate aminotransferase in the serum [245]. A positive correlation was also noted between ALT and H_2_O_2_ and the oxidative stress index in plasma of chronic hepatitis B carriers [248]. In contrast, patients with elevated aminotransferases exhibit lower α-tocopherol concentrations in serum compared to patients with persistently normal aminotransferases (PNAT) [314].

The mechanisms by which HBV-derived ROS stimulate inflammation have been poorly studied (Figure 5). The only available data were obtained in *in vitro* systems based on HBx overexpression. It was shown that HBx activates proinflammatory NF-κB signaling [260]. NF-κB is induced by several HBV-driven mechanisms, including generation of ROS in mitochondria [260], suppression of multiple cytoplasmic inhibitors of rel-related proteins [315] as well as of selenoprotein P, leading to enhanced lipid peroxidation [302]. In turn, NF-κB activation leads to the induction of proinflammatory cytokines, including TNFα [302, 316, 317], lymphotoxin-α [318], IL-1β [316, 319], and IL-6 [320], as well as interferon γ [321] and a chemokine interferon-gamma inducible protein 10 (IP-10) [322]. The induction of proinflammatory cytokines (IL-6, TNF-α) in HBx-expressing cells is augmented by a high fat diet/fatty acids through the efflux of calcium ions from ER stores and consequent overproduction of ROS [323]. Furthermore, simultaneously with the induction of the proinflammatory cytokines, HBV antigens, namely HBx, repress the expression of anti-inflammatory cytokines [316].

Also, HBx, in a ROS-dependent manner, induces the expression of cyclooxygenase 2 [266]. COX2 is rapidly expressed in several cell types in response to growth factors, cytokines, and pro-inflammatory molecules and is the dominant source of prostaglandin formation in acute and chronic inflammatory conditions [324]. COX2 induction by HBx may also be NF-κB-mediated since NF-κB is known to control COX2 expression (for example [325]).

Glutathione-S-transferase P1 (GSTP1) is an important phase II enzyme that can protect cells from oxidative stress in various human cancers. In CHB patients who progress to HCC, GST-P1 expression decreases compared to patients in earlier stages of the disease, and its level correlates positively with MDA and xanthine oxidase expression and negatively with GSH levels [326]. Specifically, acute-on-chronic hepatitis B patients are characterized by frequent GSTP1 (hyper)methylation, leading to the absence of expression of this protein [327]. GSTP1 (hyper)methylation is a hallmark of carcinogenesis and the most common molecular alteration in human cancer [328, 329]. In HBV infection as well, high GSTP1 methylation status predicts a poor prognosis [327].

Although HBV (HBx)-triggered oxidative stress may lead to cell death [269, 330], solid data have been accumulated suggesting that HBV can also confer protection against exogenous ROS, such as hydrogen peroxide [331–334]. This is achieved by several routes, including (i) activation of the Nrf2/ARE pathway [287] and subsequent induction or augmentation of expression of liver regeneration (ALR) protein [335]; (ii) hypermethylation of the p16^INK4a^ promoter, leading to alterations in the induction of a senescence p16^INK4a^ regulator with subsequent prevention of cell cycle arrest [332]; and (iii) induction of the forkhead transcription factor FOXO4 [333]. Forkhead transcription factors of the FOXO class have been implicated in cellular protection against oxidative stress via the transcriptional regulation of manganese superoxide dismutase (SOD2) and catalase gene expression [336]. Altogether, HBx expression in liver cells causes oxidative stress and at the same time boosts the survival of the “stressed” cells [331], which serves for HBV persistence in the inflamed cell environment.

HBV-induced oxidative stress promotes liver carcinogenesis. Development of liver tumors is thought to be driven by several mechanisms, including modulation of signaling pathways, resistance to ROS-triggered apoptosis, promotion of genome instability and metabolic adaptation of the infected cells. Indeed, increased O_2_^•−^ and MDA levels are present in HCC tissues compared to non-tumor liver samples and concentrations of oxidized glutathione are elevated in the blood of chronic hepatitis C carriers with HCC [337].

A recent study of Yuan *et al.* revealed that HBV-induced carcinogenesis is promoted via ROS-mediated induction of IL-6 and concomitant activation of STAT3 transcription factor as well as by induction of Snai1 and subsequent down-regulation of a suppressor of cytokine signaling 3 (SOCS3), an inhibitor of IL-6/STAT3 pathway [246]. These data obtained in laboratory models were verified in xenograft mice model as well as by notion that a life span of chronic HBV carriers is lower in patients with higher expression of Snail and with lower expression of SOCS3 [246].

ROS are known to be harmful for both the genomic and the mitochondrial DNA of the host cell. Indeed, HBV [255] and, in particular, its HBx [261] and PreS [281] proteins induce an increased oxidation of DNA. The latter is accompanied by an enhanced expression of OGG1, a DNA repair enzyme frequently (over)expressed in HCC tissue [281, 298]. At the same time, HBV decreases the expression of apurinic/apyrimidinic endonuclease 1 (APE-1) [338]. A combination of these two processes explains an observed correlation between the oxidative DNA damage and hepatocyte immortalization [204], Even an occult HBV infection appears to trigger oxidative stress and DNA damage not just in the liver but also in the peripheral blood lymphocytes. The latter may contribute to immune dysfunctions, which in turn aggravate liver damage and increase the rate of HCC occurrence [249]. One can speculate that DNA oxidation and concomitant removal of oxidized nucleic bases in HBV-infected cells is compensated by a defective repair of the oxidative damage, with a concomitant accumulation of single-strand breaks. This hypothesis is supported by data which indicate that HVB infection leads to activation of the ATM-Chk2 pathway [339] responsible for the repair of double-strand breaks, DNA damage response resulting in an increased genomic instability [340].

Indeed, integration of HBV genomes is often observed in HCC tissues compared to surrounding non-tumor tissue [341–343]. Oxidative stress and H_2_O_2,_ in particular, enhance the integration of both HBV [311] and duck hepatitis B virus [305]. Interestingly, the preferred sites of HBV DNA integration appear to be repeat elements such as Alu and minisatellite sequences, while integration into cellular genes, thought to be important for the regulation of cell division, is rare [344]. Furthermore, integration often modifies genes controlling carcinogenesis and triggers cancer-related signaling pathways [345]. Namely, HBV induces a number of procarcinogenic signaling cascades *via* ROS. HBV-induced oxidative stress in HCC tissues enhances methylation of the promoter of runt-related transcription factor 3 (RUNX3) [346]. RUNX3 is a protein whose inactivation has been implicated in the development of various solid tumors [347] thus prompting speculation that methylation of its promoter may be a key event in HBV-associated pathogenesis. HBV also causes a ROS-dependent translocation of Raf-1 kinase to mitochondria [348]. Raf-1 kinase represents a proto-oncogene responsible for activation of ERK1/2 mitogen-activated protein kinases [349]. In addition, HBx triggers oxidation of a tumor suppressor phosphatase and tensin homolog protein (PTEN), leading to its inactivation and subsequently to permanent activation of the PI3K/AKT pathway, with potentially important metabolic consequences on lipid homeostasis [350, 351].

HBx protein can also alter carbohydrate fluxes. Via activation of Nrf2, HBx is known to induce glucose-6-phosphate dehydrogenase (G6PD) [291] and to increase levels of ATP, NADPH and fatty acid oxidation, which may promote cellular survival by conferring on the infected cells a resistance to glucose deprivation [352].

Analysis of lipid metabolites in HBx transgenic mice showed that arachidonate 5-lipoxygenase, lipoprotein lipase, fatty acid binding protein 4, 1-acylglycerol-3-phosphate O-acyltransferase 9, and apolipoprotein A-IV expression are all induced [353]. Furthermore, HBx-transgenic mice exhibit elevated levels of cholesterol and triglycerides, as well as activated expression of the key transcription factors in lipid homeostasis [353], such as the prolipogenic sterol regulatory element binding protein 1 (SREBP-1), liver X receptor [354], C/EBP1 and peroxisome proliferator-activated receptor γ (PPARγ) [355]. In turn, liver PPAR-γ and SREBP-1c up-regulation in parallel with PPAR-α down-regulation enhance *de novo* lipogenesis and reduce fatty acid oxidation [356]. On the long term the consequences of this is an onset of steatosis and related long-chain polyunsaturated fatty acid n-3 (LCPUFA n-3) depletion, insulin resistance, hypoadiponectinemia, and endoplasmic reticulum stress. Although no data are yet available on the role of ROS in the activation of these transcription factors, the activation of CREBP1 in a ROS-dependent fashion by HCV has been already demonstrated [357].

As a result (of the induction and promotion of continuous oxidative stress), disease progression in chronic hepatitis B correlates to a number of polymorphisms in the stress response genes and enzymes described above. Polymorphisms correlating with disease progression and outcome occur in CYP2E1, HOGG1, and XRCC1 genes [358]. CYP2E1 and HOGG1 polymorphisms independently correlate with the development of fibrosis, whereas the c2 variant of CYP2E1 correlates with the severity of liver disease and histology activity index. Also, certain alleles (rs769217 T) in the gene encoding catalase, an endogenous antioxidant enzyme involved in ROS neutralizing pathways, appear to predispose to the development of chronic hepatitis B, and HBV-induced liver cirrhosis and HCC [359].

Altogether, these data suggest that HBV infection induces a series of metabolic changes, most of which serve to aggravate the oxidative stress response and at the same time provide the infected cells with instruments to survive it, thus paving the way to cell transformation.

#### HCC incidence in patients with chronic hepatitis B or C: effect of antiviral drugs

Since HBV and HCV are oncogenic viruses, treating and eliminating the infection should prevent liver cancer. Indeed, viral suppression or control in chronic hepatitis B patients substantially reduces HCC risk [360–363]. As an example, treatment of patients in Korea reduced the incidence of HCC during a 5 year follow by 7.1% to 58.8% depending on the treatment regimen [360]. Treatment of patients with persistently normal or moderately elevated liver enzymes with nucleos(t)ide analogues resulted in a significantly lower risk of HCC incidence [364]. In patients with cirrhosis, 4-year treatment with entecavir led to 60% reduction in HCC occurrence [365]. Antiviral therapy was also shown to reduce risk of HCC occurrence for patients with compensated cirrhosis and low but detectable viral load [366] or even with decompensated cirrhosis [367]. Noteworthy, in most studies entecavir displayed higher ability to prevent liver cancer compared to other drugs such as lamivudine or adefovir [368], although this difference was not statistically significant in all studies [369, 370]. Pegylated interferon α treatment was reported to even better reduce HCC incidence than the nucleos(t)ide drugs [371]. However, none of these treatments could totally reduce the incidence of liver cancer back to baseline (for example, [367, 372]). Overall HCC incidence rates in patients with incomplete viral control remain elevated compared to patients with inactive stage disease [373].

An important question is whether it is worth to treat HBV infection in patients who already developed tumors in the liver. In liver cancer patients presence of HBV profoundly worsens prognosis [374]. Moreover, the effect of the infection is virus titer-dependent: the cut-off values for event-free survival and overall survival were reported to be 10,100 and 12,800 IU/mL, respectively, requiring treatment of the infection prior to removal of the tumor by hepatectomy [374]. However, a study of a cohort in Hong Kong revealed that treatment of such patients with nucleos(t)ide analogues reduces risks of HCC incidence if it starts after and not before surgical removal of a tumor [375]. Interestingly, no differences were found between effects of lamivudin and entecavir [375]. Treatment of chronic hepatitis B also reduced risks of HCC recurrence after radiofrequency ablation [376].

Our knowledge on prevention of HCC and reversal of fibrosis in chronic hepatitis C has much advanced with the recent availability of potent antiviral drugs (see Lee *at al.* [377] for excellent review). An important number of clinical investigations show the benefits of a sustained virological response (SVR) independently of the fibrosis stage [378]. These benefits include reduced levels of complications related to end-stage liver disease, reduced mortality and improved quality of life [379]. Importantly, upon elimination of the virus, fibrosis is reversible in up to 87-93% of patients, only in a minority of patients fibrosis does not regress or even progress despite successful antiviral treatment [380, 381]. However, histological analysis of the liver in HCV patients pre- and post-SVR has shown, that albeit a significant reduction in the Metavir score and a normalization of liver functions, hepatic stellate cell activation and inflammatory activity did not reverse or even become more severe over time in these patients, and this occurred independently of the post-treatment fibrosis stage [382]. Further histological analysis will be necessary to validate and extend these data on fibrosis reversal. While the overall risk of HCC decreases upon SVR in chronic hepatitis C patients, it does not completely drop back to baseline and particularly patients with advanced fibrosis remain at elevated risk for HCC for at least 8 to 10 years after SVR [243, 383–388]. HCC risk factors include pretreatment fibrosis score, but also age, steatosis/genotype 3, gender, diabetes and alcohol consumption [377, 388]. In patients who had eradicated HCC prior to antiviral treatment, achievement of SVR does not prevent recurrence of liver cancer, especially in the short term [387]. However, treatment of the virus before tumor resection in many cases prevents recurrence of the infection after liver transplantation [389]. Several antiretroviral drugs trigger oxidative stress, however to our knowledge the effect of anti-HBV/HCV agents on ROS production has never been investigated. Recently Reig *et al.* noted higher rates of HCC recurrence in patients who received treatment with all-oral antiviral drugs, at least compared to treatment with pegylated interferon [390], and these results were supported by other groups (for example, see [391]). Other groups did not support differences between HCC incidence in patients with SVR following interferon-based or all-oral antivirals [392, 393]. Nevertheless, safety of direct acting antivirals and their effect on redox systems has yet to be investigated.

In neither HBV not HCV infection occurrence of hepatocellular carcinoma has been directly correlated to alterations in redox parameters. Treatment of chronic HBV infection with nucleos(t)ide analogues is accompanied by reduction of MDA levels and increase in expression of antioxidant defense enzymes [394]. In case of chronic hepatitis C, interferon α-based treatment leads to reduction of elevated levels of oxidative stress markers such as MDA [53, 58, 395], or oxysterol [60], as well as restores depletion of antioxidant status in patients’ serum [58, 395]. Nanba *et al.* reported a correlation between levels of 8-oxoG and HCC incidence in CHC patients treated with a combination of pegylated interferon and ribavirin [198]. Moreover, in patients who fail to achieve SVR MDA levels exceed the levels for SVR patients thus correlating to the HCC occurrence rates for both groups [396]. These data warrant a more detailed investigation of the role of oxidative stress in the neoplastic transformation process in order to be able to develop therapeutic means towards the prevention of HCC.

## CONCLUSIONS AND FUTURE PERSPECTIVES

Viruses disturb the physiological balance between ROS producing and ROS eliminating pathways in a normal cell because they need to adopt and optimize the cellular environment for their replication. Both HBV and HCV infections are characterized by accumulation of a similar spectrum of oxidative stress markers in liver and blood of the patients. Overall the underlying molecular mechanisms have been well studied for HCV, but remain much more obscure for HBV due to the absence of efficient in vitro infection systems. Both viruses trigger ROS production in the infected hepatocytes due to mitochondrial dysfunction and unfolded protein response. In case of HCV, additional sources of ROS were identified, such as NADPH oxidases, CYP2E1 and Ero1a, but their involvement in HBV-induced oxidative stress has yet to be studied. Besides, most studies of mechanisms of ROS production in HBV-infected cells were carried out on cell lines overexpressing individual protein of the virus, thus requiring verification of the obtained results in infectious models. Both, HCV and HBV infections were also shown to affect expression of antioxidant defense enzymes. However, most of these results are inconsistent between various groups and models used. A further particular gap in the field is the almost complete absence of data on the impact of HBV/HCV on the ROS scavenging protein families of peroxiredoxins and glutathione peroxidases and the respective roles of these latter families in the life cycle of these viruses. For both, HBC and HCV, there is a number of findings indicating that ROS can affect various stages of their life cycle. For example, the fusiogenicity of HCV virion membranes is inhibited by lipid peroxidation and viral replication is sensitive to peroxide levels. However, with a few exceptions the underlying molecular mechanisms remain unknown. HBV and HCV-triggered ROS production was also shown to promote expression and secretion of proinflammatory cytokines and to drive liver inflammation. There are also some data showing that ROS contribute to neoplastic transformation of the host cell by various mechanisms: they interfere with DNA reparation systems responsible for removal of oxidized DNA bases as well as by other mechanisms. ROS trigger metabolic changes and in particular glycolytic adaptation and enhanced lipid biosynthesis, the latter promoting steatosis which in turn contributes to hepatocarcinogenesis. HCV associated ROS were also shown to block proapoptotic pathways and to promote cell cycle progression, both of which are crucial for cell transformation. In conclusion, we need a better and more detailed understanding of how HBV and HCV alter ROS producing and ROS scavenging events and to assess their impact on fibrosis and neoplastic transformation. These studies will be important, as targeting the redox homeostasis therapeutically can have potential anti-inflammatory and anti-fibrotic as well as antiviral effects. However, these approaches need to go beyond the simple use of antioxidants, as in the context of patients, where neoplastic transformation events have already occurred, antioxidants may have procarcinogenic effects.

## References

[1] 2014 Cancer. Fact sheet No 297.

[2] 2014 The top 10 causes of death. Fact sheet No 310.

[3] Torre LA, Bray F, Siegel RL, Ferlay J, Lortet-Tieulent J, Jemal A (2015). Global cancer statistics, 2012. CA Cancer J Clin.

[4] Torre LA, Siegel RL, Ward EM, Jemal A (2016). Global Cancer Incidence and Mortality Rates and Trends--An Update. Cancer Epidemiol Biomarkers Prev.

[5] Ryerson AB, Eheman CR, Altekruse SF, Ward JW, Jemal A, Sherman RL, Henley SJ, Holtzman D, Lake A, Noone AM, Anderson RN, Ma J, Ly KN (2016). Annual Report to the Nation on the Status of Cancer, 1975-2012, featuring the increasing incidence of liver cancer. Cancer.

[6] Ferlay J, Soerjomataram I, Dikshit R, Eser S, Mathers C, Rebelo M, Parkin DM, Forman D, Bray F (2015). Cancer incidence and mortality worldwide: sources, methods and major patterns in GLOBOCAN 2012. Int J Cancer.

[7] Sartorius K, Sartorius B, Aldous C, Govender PS, Madiba TE (2015). Global and country underestimation of hepatocellular carcinoma (HCC) in 2012 and its implications. Cancer Epidemiol.

[8] Plummer M, de Martel C, Vignat J, Ferlay J, Bray F, Franceschi S (2016). Global burden of cancers attributable to infections in 2012: a synthetic analysis. Lancet Glob Health.

[9] de Martel C, Maucort-Boulch D, Plummer M, Franceschi S (2015). World-wide relative contribution of hepatitis B and C viruses in hepatocellular carcinoma. Hepatology.

[10] Murray CJ, Barber RM, Foreman KJ, Abbasoglu Ozgoren A, Abd-Allah F, Abera SF, Aboyans V, Abraham JP, Abubakar I, Abu-Raddad LJ, Abu-Rmeileh NM, Achoki T, Ackerman IN (2015). Global, regional, and national disability-adjusted life years (DALYs) for 306 diseases and injuries and healthy life expectancy (HALE) for 188 countries, 1990-2013: quantifying the epidemiological transition. Lancet.

[11] Omland LH, Jepsen P, Krarup H, Christensen PB, Weis N, Nielsen L, Obel N, Sorensen HT, Stuver SO (2012). Liver cancer and non-Hodgkin lymphoma in hepatitis C virus-infected patients: results from the DANVIR cohort study. Int J Cancer.

[12] Omland LH, Krarup H, Jepsen P, Georgsen J, Harritshoj LH, Riisom K, Jacobsen SE, Schouenborg P, Christensen PB, Sorensen HT, Obel N (2010). Mortality in patients with chronic and cleared hepatitis C viral infection: a nationwide cohort study. J Hepatol.

[13] Braks RE, Ganne-Carrie N, Fontaine H, Paries J, Grando-Lemaire V, Beaugrand M, Pol S, Trinchet JC (2007). Effect of sustained virological response on long-term clinical outcome in 113 patients with compensated hepatitis C-related cirrhosis treated by interferon alpha and ribavirin. World J Gastroenterol.

[14] Bruno S, Stroffolini T, Colombo M, Bollani S, Benvegnu L, Mazzella G, Ascione A, Santantonio T, Piccinino F, Andreone P, Mangia A, Gaeta GB, Persico M (2007). Sustained virological response to interferon-alpha is associated with improved outcome in HCV-related cirrhosis: a retrospective study. Hepatology.

[15] Terrault NA, Bzowej NH, Chang KM, Hwang JP, Jonas MM, Murad MH (2016). AASLD guidelines for treatment of chronic hepatitis B. Hepatology.

[16] Bartosch B, Thimme R, Blum HE, Zoulim F (2009). Hepatitis C virus-induced hepatocarcinogenesis. J Hepatol.

[17] Ivanov AV, Bartosch B, Smirnova OA, Isaguliants MG, Kochetkov SN (2013). HCV and oxidative stress in the liver. Viruses.

[18] Gutowski M, Kowalczyk S (2013). A study of free radical chemistry: their role and pathophysiological significance. Acta Biochim Pol.

[19] Augusto O, Miyamoto S, Pantopoulos K, Schipper HM (2011). Oxygen Radicals and Related Species. Principles of Free Radical Biomedicine.

[20] Lipinski B (2011). Hydroxyl radical and its scavengers in health and disease. Oxid Med Cell Longev, 2011.

[21] Cadet J, Wagner JR (2014). Oxidatively generated base damage to cellular DNA by hydroxyl radical and one-electron oxidants: similarities and differences. Arch Biochem Biophys.

[22] Winterbourn CC (2008). Reconciling the chemistry and biology of reactive oxygen species. Nat Chem Biol.

[23] D’Autreaux B, Toledano MB (2007). ROS as signalling molecules: mechanisms that generate specificity in ROS homeostasis. Nat Rev Mol Cell Biol.

[24] Dynowski M, Schaaf G, Loque D, Moran O, Ludewig U (2008). Plant plasma membrane water channels conduct the signalling molecule H2O2. Biochem J.

[25] Veal EA, Day AM, Morgan BA (2007). Hydrogen peroxide sensing and signaling. Mol Cell.

[26] Forman HJ, Davies KJ, Ursini F (2014). How do nutritional antioxidants really work: nucleophilic tone and para-hormesis versus free radical scavenging in vivo. Free Radic Biol Med.

[27] Aleksunes LM, Manautou JE (2007). Emerging role of Nrf2 in protecting against hepatic and gastrointestinal disease. Toxicol Pathol.

[28] Zelko IN, Mariani TJ, Folz RJ (2002). Superoxide dismutase multigene family: a comparison of the CuZn-SOD (SOD1), Mn-SOD (SOD2), and EC-SOD (SOD3) gene structures, evolution, and expression. Free Radic Biol Med.

[29] Perkins A, Nelson KJ, Parsonage D, Poole LB, Karplus PA (2015). Peroxiredoxins: guardians against oxidative stress and modulators of peroxide signaling. Trends Biochem Sci.

[30] Brigelius-Flohe R, Maiorino M (2013). Glutathione peroxidases. Biochim Biophys Acta.

[31] Cox AG, Winterbourn CC, Hampton MB (2010). Mitochondrial peroxiredoxin involvement in antioxidant defence and redox signalling. Biochem J.

[32] Aoyama K, Nakaki T (2015). Glutathione in Cellular Redox Homeostasis: Association with the Excitatory Amino Acid Carrier 1 (EAAC1). Molecules.

[33] Wedemeyer H, Dore GJ, Ward JW (2015). Estimates on HCV disease burden worldwide - filling the gaps. J Viral Hepat.

[34] Rosen HR, Gretch DR (1999). Hepatitis C virus: current understanding and prospects for future therapies. Mol Med Today.

[35] Lemon SM, Walker CM, Alter MJ, Yi M-K, Knipe DM, Howley PM (2007). Hepatitis C virus. Fields Virology.

[36] Kawaguchi Y, Mizuta T (2014). Interaction between hepatitis C virus and metabolic factors. World J Gastroenterol.

[37] de Sanjose S, Benavente Y, Vajdic CM, Engels EA, Morton LM, Bracci PM, Spinelli JJ, Zheng T, Zhang Y, Franceschi S, Talamini R, Holly EA, Grulich AE (2008). Hepatitis C and non-Hodgkin lymphoma among 4784 cases and 6269 controls from the International Lymphoma Epidemiology Consortium. Clin Gastroenterol Hepatol.

[38] Schollkopf C, Smedby KE, Hjalgrim H, Rostgaard K, Panum I, Vinner L, Chang ET, Glimelius B, Porwit A, Sundstrom C, Hansen M, Adami HO, Melbye M (2008). Hepatitis C infection and risk of malignant lymphoma. Int J Cancer.

[39] Sansonno D, De Vita S, Iacobelli AR, Cornacchiulo V, Boiocchi M, Dammacco F (1998). Clonal analysis of intrahepatic B cells from HCV-infected patients with and without mixed cryoglobulinemia. J Immunol.

[40] Vallat L, Benhamou Y, Gutierrez M, Ghillani P, Hercher C, Thibault V, Charlotte F, Piette JC, Poynard T, Merle-Beral H, Davi F, Cacoub P (2004). Clonal B cell populations in the blood and liver of patients with chronic hepatitis C virus infection. Arthritis Rheum.

[41] Zeisel MB, Fofana I, Fafi-Kremer S, Baumert TF (2011). Hepatitis C virus entry into hepatocytes: molecular mechanisms and targets for antiviral therapies. J Hepatol.

[42] Binder M, Sulaimanov N, Clausznitzer D, Schulze M, Huber CM, Lenz SM, Schloder JP, Trippler M, Bartenschlager R, Lohmann V, Kaderali L (2013). Replication vesicles are load- and choke-points in the hepatitis C virus lifecycle. PLoS Pathog.

[43] Bartenschlager R, Penin F, Lohmann V, Andre P (2011). Assembly of infectious hepatitis C virus particles. Trends Microbiol.

[44] Valgimigli L, Valgimigli M, Gaiani S, Pedulli GF, Bolondi L (2000). Measurement of oxidative stress in human liver by EPR spin-probe technique. Free Radic Res.

[45] Valgimigli M, Valgimigli L, Trere D, Gaiani S, Pedulli GF, Gramantieri L, Bolondi L (2002). Oxidative stress EPR measurement in human liver by radical-probe technique. Correlation with etiology, histology and cell proliferation. Free Radic Res.

[46] Kitada T, Seki S, Iwai S, Yamada T, Sakaguchi H, Wakasa K (2001). In situ detection of oxidative DNA damage, 8-hydroxydeoxyguanosine, in chronic human liver disease. J Hepatol.

[47] Vendemiale G, Grattagliano I, Portincasa P, Serviddio G, Palasciamo G, Altomare E (2001). Oxidative stress in symptom-free HCV carriers: relation with ALT flare-up. Eur J Clin Invest.

[48] Barbaro G, Di Lorenzo G, Ribersani M, Soldini M, Giancaspro G, Bellomo G, Belloni G, Grisorio B, Barbarini G (1999). Serum ferritin and hepatic glutathione concentrations in chronic hepatitis C patients related to the hepatitis C virus genotype. J Hepatol.

[49] Rahman I, Marwick J, Kirkham P (2004). Redox modulation of chromatin remodeling: impact on histone acetylation and deacetylation, NF-kappaB and pro-inflammatory gene expression. Biochem Pharmacol.

[50] Zuwala-Jagiello J, Warwas M, Pazgan-Simon M (2012). Ischemia-modified albumin (IMA) is increased in patients with chronic hepatitis C infection and related to markers of oxidative stress and inflammation. Acta Biochim Pol.

[51] De Maria N, Colantoni A, Fagiuoli S, Liu GJ, Rogers BK, Farinati F, Van Thiel DH, Floyd RA (1996). Association between reactive oxygen species and disease activity in chronic hepatitis C. Free Radic Biol Med.

[52] Osman HG, Gabr OM, Lotfy S, Gabr S (2007). Serum levels of bcl-2 and cellular oxidative stress in patients with viral hepatitis. Indian J Med Microbiol.

[53] Serejo F, Emerit I, Filipe PM, Fernandes AC, Costa MA, Freitas JP, de Moura MC (2003). Oxidative stress in chronic hepatitis C: the effect of interferon therapy and correlation with pathological features. Can J Gastroenterol.

[54] Emerit I, Serejo F, Filipe P, Alaoui Youssefi A, Fernandes A, Costa A, Freitas J, Ramalho F, Baptista A, Carneiro de Moura M (2000). Clastogenic factors as biomarkers of oxidative stress in chronic hepatitis C. Digestion.

[55] Boya P, de la Pena A, Beloqui O, Larrea E, Conchillo M, Castelruiz Y, Civeira MP, Prieto J (1999). Antioxidant status and glutathione metabolism in peripheral blood mononuclear cells from patients with chronic hepatitis C. J Hepatol.

[56] Yadav D, Hertan HI, Schweitzer P, Norkus EP, Pitchumoni CS (2002). Serum and liver micronutrient antioxidants and serum oxidative stress in patients with chronic hepatitis C. Am J Gastroenterol.

[57] Venturini D, Simao AN, Barbosa DS, Lavado EL, Narciso VE, Dichi I, Dichi JB (2010). Increased oxidative stress, decreased total antioxidant capacity, and iron overload in untreated patients with chronic hepatitis C. Dig Dis Sci.

[58] Levent G, Ali A, Ahmet A, Polat EC, Aytac C, Ayse E, Ahmet S (2006). Oxidative stress and antioxidant defense in patients with chronic hepatitis C patients before and after pegylated interferon alfa-2b plus ribavirin therapy. J Transl Med.

[59] Huang X, Liang H, Fan X, Zhu L, Shen T (2016). Liver Damage in Patients with HCV/HIV Coinfection Is Linked to HIV-Related Oxidative Stress. Oxid Med Cell Longev.

[60] Ikegami T, Honda A, Miyazaki T, Kohjima M, Nakamuta M, Matsuzaki Y (2014). Increased serum oxysterol concentrations in patients with chronic hepatitis C virus infection. Biochem Biophys Res Commun.

[61] Sumida Y, Nakashima T, Yoh T, Nakajima Y, Ishikawa H, Mitsuyoshi H, Sakamoto Y, Okanoue T, Kashima K, Nakamura H, Yodoi J (2000). Serum thioredoxin levels as an indicator of oxidative stress in patients with hepatitis C virus infection. J Hepatol.

[62] Nakashima T, Sumida Y, Yoh T, Kakisaka Y, Nakajima Y, Ishikawa H, Mitsuyoshi H, Kashima K, Nakamura H, Yodoi J (2000). Thioredoxin levels in the sera of untreated viral hepatitis patients and those treated with glycyrrhizin or ursodeoxycholic acid. Antioxid Redox Signal.

[63] Cardin R, Saccoccio G, Masutti F, Bellentani S, Farinati F, Tiribelli C (2001). DNA oxidative damage in leukocytes correlates with the severity of HCV-related liver disease: validation in an open population study. J Hepatol.

[64] Farinati F, Cardin R, Degan P, De Maria N, Floyd RA, Van Thiel DH, Naccarato R (1999). Oxidative DNA damage in circulating leukocytes occurs as an early event in chronic HCV infection. Free Radic Biol Med.

[65] Jain SK, Pemberton PW, Smith A, McMahon RF, Burrows PC, Aboutwerat A, Warnes TW (2002). Oxidative stress in chronic hepatitis C: not just a feature of late stage disease. J Hepatol.

[66] Bhargava A, Raghuram GV, Pathak N, Varshney S, Jatawa SK, Jain D, Mishra PK (2011). Occult hepatitis C virus elicits mitochondrial oxidative stress in lymphocytes and triggers PI3-kinase-mediated DNA damage response. Free Radic Biol Med.

[67] Bauerle J, Laguno M, Mauss S, Mallolas J, Murillas J, Miquel R, Schmutz G, Setzer B, Gatell JM, Walker UA (2005). Mitochondrial DNA depletion in liver tissue of patients infected with hepatitis C virus: contributing effect of HIV infection?. HIV Med.

[68] Choudhury M, Park PH, Jackson D, Shukla SD (2010). Evidence for the role of oxidative stress in the acetylation of histone H3 by ethanol in rat hepatocytes. Alcohol.

[69] Higgs MR, Chouteau P, Lerat H (2014). ‘Liver let die’: oxidative DNA damage and hepatotropic viruses. J Gen Virol.

[70] Higgs MR, Lerat H, Pawlotsky JM (2013). Hepatitis C virus-induced activation of beta-catenin promotes c-Myc expression and a cascade of pro-carcinogenetic events. Oncogene.

[71] Capone F, Guerriero E, Sorice A, Maio P, Colonna G, Castello G, Costantini S (2012). Characterization of metalloproteinases, oxidative status and inflammation levels in the different stages of fibrosis in HCV patients. Clin Biochem.

[72] Look MP, Gerard A, Rao GS, Sudhop T, Fischer HP, Sauerbruch T, Spengler U (1999). Interferon/antioxidant combination therapy for chronic hepatitis C--a controlled pilot trial. Antiviral Res.

[73] Swietek K, Juszczyk J (1997). Reduced glutathione concentration in erythrocytes of patients with acute and chronic viral hepatitis. J Viral Hepat.

[74] Ivanov AV, Smirnova OA, Ivanova ON, Masalova OV, Kochetkov SN, Isaguliants MG (2011). Hepatitis C virus proteins activate NRF2/ARE pathway by distinct ROS-dependent and independent mechanisms in HUH7 cells. PLoS One.

[75] Korenaga M, Wang T, Li Y, Showalter LA, Chan T, Sun J, Weinman SA (2005). Hepatitis C virus core protein inhibits mitochondrial electron transport and increases reactive oxygen species (ROS) production. J Biol Chem.

[76] Li Y, Boehning DF, Qian T, Popov VL, Weinman SA (2007). Hepatitis C virus core protein increases mitochondrial ROS production by stimulation of Ca2+ uniporter activity. Faseb J.

[77] Okuda M, Li K, Beard MR, Showalter LA, Scholle F, Lemon SM, Weinman SA (2002). Mitochondrial injury, oxidative stress, and antioxidant gene expression are induced by hepatitis C virus core protein. Gastroenterology.

[78] Otani K, Korenaga M, Beard MR, Li K, Qian T, Showalter LA, Singh AK, Wang T, Weinman SA (2005). Hepatitis C virus core protein, cytochrome P450 2E1, and alcohol produce combined mitochondrial injury and cytotoxicity in hepatoma cells. Gastroenterology.

[79] Pal S, Polyak SJ, Bano N, Qiu WC, Carithers RL, Shuhart M, Gretch DR, Das A (2010). Hepatitis C virus induces oxidative stress, DNA damage and modulates the DNA repair enzyme NEIL1. J Gastroenterol Hepatol.

[80] Garcia-Mediavilla MV, Sanchez-Campos S, Gonzalez-Perez P, Gomez-Gonzalo M, Majano PL, Lopez-Cabrera M, Clemente G, Garcia-Monzon C, Gonzalez-Gallego J (2005). Differential contribution of hepatitis C virus NS5A and core proteins to the induction of oxidative and nitrosative stress in human hepatocyte-derived cells. J Hepatol.

[81] Ming-Ju H, Yih-Shou H, Tzy-Yen C, Hui-Ling C (2011). Hepatitis C virus E2 protein induce reactive oxygen species (ROS)-related fibrogenesis in the HSC-T6 hepatic stellate cell line. J Cell Biochem.

[82] Bureau C, Bernad J, Chaouche N, Orfila C, Beraud M, Gonindard C, Alric L, Vinel JP, Pipy B (2001). Nonstructural 3 protein of hepatitis C virus triggers an oxidative burst in human monocytes via activation of NADPH oxidase. J Biol Chem.

[83] Li S, Ye L, Yu X, Xu B, Li K, Zhu X, Liu H, Wu X, Kong L (2009). Hepatitis C virus NS4B induces unfolded protein response and endoplasmic reticulum overload response-dependent NF-kappaB activation. Virology.

[84] Gong G, Waris G, Tanveer R, Siddiqui A (2001). Human hepatitis C virus NS5A protein alters intracellular calcium levels, induces oxidative stress, and activates STAT-3 and NF-kappa B. Proc Natl Acad Sci U S A.

[85] Wang T, Campbell RV, Yi MK, Lemon SM, Weinman SA (2010). Role of Hepatitis C virus core protein in viral-induced mitochondrial dysfunction. J Viral Hepat.

[86] Kang SM, Kim SJ, Kim JH, Lee W, Kim GW, Lee KH, Choi KY, Oh JW (2009). Interaction of hepatitis C virus core protein with Hsp60 triggers the production of reactive oxygen species and enhances TNF-alpha-mediated apoptosis. Cancer Lett.

[87] Piccoli C, Scrima R, Quarato G, D’Aprile A, Ripoli M, Lecce L, Boffoli D, Moradpour D, Capitanio N (2007). Hepatitis C virus protein expression causes calcium-mediated mitochondrial bioenergetic dysfunction and nitro-oxidative stress. Hepatology.

[88] Hoppe UC (2010). Mitochondrial calcium channels. FEBS Lett.

[89] Patergnani S, Suski JM, Agnoletto C, Bononi A, Bonora M, De Marchi E, Giorgi C, Marchi S, Missiroli S, Poletti F, Rimessi A, Duszynski J, Wieckowski MR (2011). Calcium signaling around Mitochondria Associated Membranes (MAMs). Cell Commun Signal.

[90] Boulant S, Montserret R, Hope RG, Ratinier M, Targett-Adams P, Lavergne JP, Penin F, McLauchlan J (2006). Structural determinants that target the hepatitis C virus core protein to lipid droplets. J Biol Chem.

[91] Schwer B, Ren S, Pietschmann T, Kartenbeck J, Kaehlcke K, Bartenschlager R, Yen TS, Ott M (2004). Targeting of hepatitis C virus core protein to mitochondria through a novel C-terminal localization motif. J Virol.

[92] Williamson CD, Colberg-Poley AM (2009). Access of viral proteins to mitochondria via mitochondria-associated membranes. Rev Med Virol.

[93] Anelli T, Bergamelli L, Margittai E, Rimessi A, Fagioli C, Malgaroli A, Pinton P, Ripamonti M, Rizzuto R, Sitia R (2012). Ero1alpha regulates Ca(2+) fluxes at the endoplasmic reticulum-mitochondria interface (MAM). Antioxid Redox Signal.

[94] Seervi M, Sobhan PK, Joseph J, Ann Mathew K, Santhoshkumar TR (2013). ERO1alpha-dependent endoplasmic reticulum-mitochondrial calcium flux contributes to ER stress and mitochondrial permeabilization by procaspase-activating compound-1 (PAC-1). Cell Death Dis.

[95] Ivanov AV, Smirnova OA, Petrushanko IY, Ivanova ON, Karpenko IL, Alekseeva E, Sominskaya I, Makarov AA, Bartosch B, Kochetkov SN, Isaguliants MG (2015). HCV core protein uses multiple mechanisms to induce oxidative stress in human hepatoma Huh7 cells. Viruses.

[96] Tsutsumi T, Matsuda M, Aizaki H, Moriya K, Miyoshi H, Fujie H, Shintani Y, Yotsuyanagi H, Miyamura T, Suzuki T, Koike K (2009). Proteomics analysis of mitochondrial proteins reveals overexpression of a mitochondrial protein chaperon, prohibitin, in cells expressing hepatitis C virus core protein. Hepatology.

[97] Artal-Sanz M, Tavernarakis N (2009). Prohibitin and mitochondrial biology. Trends Endocrinol Metab.

[98] Jekabsone A, Ivanoviene L, Brown GC, Borutaite V (2003). Nitric oxide and calcium together inactivate mitochondrial complex I and induce cytochrome c release. J Mol Cell Cardiol.

[99] Machida K, Cheng KT, Sung VM, Lee KJ, Levine AM, Lai MM (2004). Hepatitis C virus infection activates the immunologic (type II) isoform of nitric oxide synthase and thereby enhances DNA damage and mutations of cellular genes. J Virol.

[100] Grivennikova VG, Vinogradov AD (2013). Mitochondrial production of reactive oxygen species. Biochemistry (Mosc).

[101] Starkov AA, Fiskum G, Chinopoulos C, Lorenzo BJ, Browne SE, Patel MS, Beal MF (2004). Mitochondrial alpha-ketoglutarate dehydrogenase complex generates reactive oxygen species. J Neurosci.

[102] Kareyeva AV, Grivennikova VG, Cecchini G, Vinogradov AD (2011). Molecular identification of the enzyme responsible for the mitochondrial NADH-supported ammonium-dependent hydrogen peroxide production. FEBS Lett.

[103] Tretter L, Adam-Vizi V (2005). Alpha-ketoglutarate dehydrogenase: a target and generator of oxidative stress. Philos Trans R Soc Lond B Biol Sci.

[104] Denton RM (2009). Regulation of mitochondrial dehydrogenases by calcium ions. Biochim Biophys Acta.

[105] Benali-Furet NL, Chami M, Houel L, De Giorgi F, Vernejoul F, Lagorce D, Buscail L, Bartenschlager R, Ichas F, Rizzuto R, Paterlini-Brechot P (2005). Hepatitis C virus core triggers apoptosis in liver cells by inducing ER stress and ER calcium depletion. Oncogene.

[106] Chan SW, Egan PA (2005). Hepatitis C virus envelope proteins regulate CHOP via induction of the unfolded protein response. Faseb J.

[107] Zheng Y, Gao B, Ye L, Kong L, Jing W, Yang X, Wu Z, Ye L (2005). Hepatitis C virus non-structural protein NS4B can modulate an unfolded protein response. J Microbiol.

[108] Malhi H, Kaufman RJ (2011). Endoplasmic reticulum stress in liver disease. J Hepatol.

[109] Pellegrino MW, Nargund AM, Haynes CM (2013). Signaling the mitochondrial unfolded protein response. Biochim Biophys Acta.

[110] Li G, Mongillo M, Chin KT, Harding H, Ron D, Marks AR, Tabas I (2009). Role of ERO1-alpha-mediated stimulation of inositol 1,4,5-triphosphate receptor activity in endoplasmic reticulum stress-induced apoptosis. J Cell Biol.

[111] Robinson LC, Marchant JS (2008). Enhanced Ca2+ leak from ER Ca2+ stores induced by hepatitis C NS5A protein. Biochem Biophys Res Commun.

[112] Dionisio N, Garcia-Mediavilla MV, Sanchez-Campos S, Majano PL, Benedicto I, Rosado JA, Salido GM, Gonzalez-Gallego J (2009). Hepatitis C virus NS5A and core proteins induce oxidative stress-mediated calcium signalling alterations in hepatocytes. J Hepatol.

[113] Smirnova OA, Ivanova ON, Bartosch B, Valuev-Ellison VT, Mukhtarov F, Kochetkov SN, Ivanov AV (2016). Hepatitis C Virus NS5A Protein Triggers Oxidative Stress by Inducing NADPH Oxidases 1 and 4 and Cytochrome P450 2E1. Oxid Med Cell Longev.

[114] Boudreau HE, Emerson SU, Korzeniowska A, Jendrysik MA, Leto TL (2009). Hepatitis C virus (HCV) proteins induce NADPH oxidase 4 expression in a transforming growth factor beta-dependent manner: a new contributor to HCV-induced oxidative stress. J Virol.

[115] de Mochel NS, Seronello S, Wang SH, Ito C, Zheng JX, Liang TJ, Lambeth JD, Choi J (2010). Hepatocyte NAD(P)H oxidases as an endogenous source of reactive oxygen species during hepatitis C virus infection. Hepatology.

[116] Sancho P, Martin-Sanz P, Fabregat I (2011). Reciprocal regulation of NADPH oxidases and the cyclooxygenase-2 pathway. Free Radic Biol Med.

[117] Spencer NY, Yan Z, Boudreau RL, Zhang Y, Luo M, Li Q, Tian X, Shah AM, Davisson RL, Davidson B, Banfi B, Engelhardt JF (2011). Control of hepatic nuclear superoxide production by glucose 6-phosphate dehydrogenase and NADPH oxidase-4. J Biol Chem.

[118] Takac I, Schroder K, Zhang L, Lardy B, Anilkumar N, Lambeth JD, Shah AM, Morel F, Brandes RP (2011). The E-loop is involved in hydrogen peroxide formation by the NADPH oxidase Nox4. J Biol Chem.

[119] Avadhani NG, Sangar MC, Bansal S, Bajpai P (2011). Bimodal targeting of cytochrome P450s to endoplasmic reticulum and mitochondria: the concept of chimeric signals. FEBS J.

[120] Lieber CS (1997). Cytochrome P-4502E1: its physiological and pathological role. Physiol Rev.

[121] Lu Y, Cederbaum AI (2008). CYP2E1 and oxidative liver injury by alcohol. Free Radic Biol Med.

[122] Nakai K, Tanaka H, Hanada K, Ogata H, Suzuki F, Kumada H, Miyajima A, Ishida S, Sunouchi M, Habano W, Kamikawa Y, Kubota K, Kita J (2008). Decreased expression of cytochromes P450 1A2, 2E1, and 3A4 and drug transporters Na+-taurocholate-cotransporting polypeptide, organic cation transporter 1, and organic anion-transporting peptide-C correlates with the progression of liver fibrosis in chronic hepatitis C patients. Drug Metab Dispos.

[123] Wen F, Abdalla MY, Aloman C, Xiang J, Ahmad IM, Walewski J, McCormick ML, Brown KE, Branch AD, Spitz DR, Britigan BE, Schmidt WN (2004). Increased prooxidant production and enhanced susceptibility to glutathione depletion in HepG2 cells co-expressing HCV core protein and CYP2E1. J Med Virol.

[124] Hiura M, Honma Y, Miyagawa K, Oe S, Shimajiri S, Mihara H, Oe M, Sato-Morita M, Katsuki Y, Harada M (2015). Alleviation mechanisms against hepatocyte oxidative stress in patients with chronic hepatic disorders. Hepatol Res.

[125] Laurindo FR, Pescatore LA, Fernandes Dde C (2012). Protein disulfide isomerase in redox cell signaling and homeostasis. Free Radic Biol Med.

[126] Ramming T, Appenzeller-Herzog C (2012). The physiological functions of mammalian endoplasmic oxidoreductin 1: on disulfides and more. Antioxid Redox Signal.

[127] Tavender TJ, Bulleid NJ (2010). Peroxiredoxin IV protects cells from oxidative stress by removing H2O2 produced during disulphide formation. J Cell Sci.

[128] Ramming T, Hansen HG, Nagata K, Ellgaard L, Appenzeller-Herzog C (2014). GPx8 peroxidase prevents leakage of H2O2 from the endoplasmic reticulum. Free Radic Biol Med.

[129] Ramming T, Okumura M, Kanemura S, Baday S, Birk J, Moes S, Spiess M, Jeno P, Berneche S, Inaba K, Appenzeller-Herzog C (2015). A PDI-catalyzed thiol-disulfide switch regulates the production of hydrogen peroxide by human Ero1. Free Radic Biol Med.

[130] Khadem Ansari MH, Omrani MD, Kheradmand F (2015). Oxidative stress response in patients infected by diverse hepatitis C virus genotypes. Hepat Mon.

[131] Carvajal-Yepes M, Himmelsbach K, Schaedler S, Ploen D, Krause J, Ludwig L, Weiss T, Klingel K, Hildt E (2011). Hepatitis C virus impairs the induction of cytoprotective Nrf2 target genes by delocalization of small Maf proteins. J Biol Chem.

[132] Burdette D, Olivarez M, Waris G (2010). Activation of transcription factor Nrf2 by hepatitis C virus induces the cell-survival pathway. J Gen Virol.

[133] Jiang Y, Bao H, Ge Y, Tang W, Cheng D, Luo K, Gong G, Gong R (2015). Therapeutic targeting of GSK3beta enhances the Nrf2 antioxidant response and confers hepatic cytoprotection in hepatitis C. Gut.

[134] Bessa SS, Mohamed Ali EM, Abd El-Wahab Ael S, Nor El-Din SA (2012). Heme oxygenase-1 mRNA expression in egyptian patients with chronic liver disease. Hepat Mon.

[135] Ghaziani T, Shan Y, Lambrecht RW, Donohue SE, Pietschmann T, Bartenschlager R, Bonkovsky HL (2006). HCV proteins increase expression of heme oxygenase-1 (HO-1) and decrease expression of Bach1 in human hepatoma cells. J Hepatol.

[136] Hou WH, Rossi L, Shan Y, Zheng JY, Lambrecht RW, Bonkovsky HL (2009). Iron increases HMOX1 and decreases hepatitis C viral expression in HCV-expressing cells. World J Gastroenterol.

[137] Jablonowska E, Wojcik K, Szymanska B, Omulecka A, Cwiklinska H, Piekarska A (2014). Hepatic HMOX1 expression positively correlates with Bach-1 and miR-122 in patients with HCV mono and HIV/HCV coinfection. PLoS One.

[138] Abdalla MY, Britigan BE, Wen F, Icardi M, McCormick ML, LaBrecque DR, Voigt M, Brown KE, Schmidt WN (2004). Down-regulation of heme oxygenase-1 by hepatitis C virus infection in vivo and by the in vitro expression of hepatitis C core protein. J Infect Dis.

[139] Chen WC, Wang SY, Chiu CC, Tseng CK, Lin CK, Wang HC, Lee JC (2013). Lucidone suppresses hepatitis C virus replication by Nrf2-mediated heme oxygenase-1 induction. Antimicrob Agents Chemother.

[140] Lee JC, Tseng CK, Young KC, Sun HY, Wang SW, Chen WC, Lin CK, Wu YH (2014). Andrographolide exerts anti-hepatitis C virus activity by up-regulating haeme oxygenase-1 via the p38 MAPK/Nrf2 pathway in human hepatoma cells. Br J Pharmacol.

[141] Sun J, Hoshino H, Takaku K, Nakajima O, Muto A, Suzuki H, Tashiro S, Takahashi S, Shibahara S, Alam J, Taketo MM, Yamamoto M, Igarashi K (2002). Hemoprotein Bach1 regulates enhancer availability of heme oxygenase-1 gene. Embo J.

[142] Yu JS, Chen WC, Tseng CK, Lin CK, Hsu YC, Chen YH, Lee JC (2016). Sulforaphane Suppresses Hepatitis C Virus Replication by Up-Regulating Heme Oxygenase-1 Expression through PI3K/Nrf2 Pathway. PLoS One.

[143] Tang W, Lazaro CA, Campbell JS, Parks WT, Katze MG, Fausto N (2007). Responses of nontransformed human hepatocytes to conditional expression of full-length hepatitis C virus open reading frame. Am J Pathol.

[144] Smirnova OA, Ivanova ON, Mukhtarov FS, Tunitskaya VL, Jansons J, Isaguliants MG, Kochetkov SN, Ivanov AV (2016). Analysis of the Domains of Hepatitis C Virus Core and NS5A Proteins that Activate the Nrf2/ARE Cascade. Acta Naturae.

[145] Brault C, Levy P, Duponchel S, Michelet M, Salle A, Pecheur EI, Plissonnier ML, Parent R, Vericel E, Ivanov AV, Demir M, Steffen HM, Odenthal M (2016). Glutathione peroxidase 4 is reversibly induced by HCV to control lipid peroxidation and to increase virion infectivity. Gut.

[146] Blackham S, Baillie A, Al-Hababi F, Remlinger K, You S, Hamatake R, McGarvey MJ (2010). Gene expression profiling indicates the roles of host oxidative stress, apoptosis, lipid metabolism, and intracellular transport genes in the replication of hepatitis C virus. J Virol.

[147] Walters KA, Syder AJ, Lederer SL, Diamond DL, Paeper B, Rice CM, Katze MG (2009). Genomic analysis reveals a potential role for cell cycle perturbation in HCV-mediated apoptosis of cultured hepatocytes. PLoS Pathog.

[148] Roulot D, Durand H, Coste T, Rautureau J, Strosberg AD, Benarous R, Marullo S (1995). Quantitative analysis of transforming growth factor beta 1 messenger RNA in the liver of patients with chronic hepatitis C: absence of correlation between high levels and severity of disease. Hepatology.

[149] Nelson DR, Gonzalez-Peralta RP, Qian K, Xu Y, Marousis CG, Davis GL, Lau JY (1997). Transforming growth factor-beta 1 in chronic hepatitis C. J Viral Hepat.

[150] Kirmaz C, Terzioglu E, Topalak O, Bayrak P, Yilmaz O, Ersoz G, Sebik F (2004). Serum transforming growth factor-beta1(TGF-beta1) in patients with cirrhosis, chronic hepatitis B and chronic hepatitis C [corrected]. Eur Cytokine Netw.

[151] Kotsiri I, Hadziyannis E, Georgiou A, Papageorgiou MV, Vlachogiannakos I, Papatheodoridis G (2016). Changes in serum transforming growth factor-beta1 levels in chronic hepatitis C patients under antiviral therapy. Ann Gastroenterol.

[152] Flores-Contreras L, Sandoval-Rodriguez AS, Mena-Enriquez MG, Lucano-Landeros S, Arellano-Olivera I, Alvarez-Alvarez A, Sanchez-Parada MG, Armendariz-Borunda J (2014). Treatment with pirfenidone for two years decreases fibrosis, cytokine levels and enhances CB2 gene expression in patients with chronic hepatitis C. BMC Gastroenterol.

[153] Abdalla MY, Ahmad IM, Spitz DR, Schmidt WN, Britigan BE (2005). Hepatitis C virus-core and non structural proteins lead to different effects on cellular antioxidant defenses. J Med Virol.

[154] Morikawa K, Gouttenoire J, Hernandez C, Dao Thi VL, Tran HT, Lange CM, Dill MT, Heim MH, Donze O, Penin F, Quadroni M, Moradpour D (2014). Quantitative proteomics identifies the membrane-associated peroxidase GPx8 as a cellular substrate of the hepatitis C virus NS3-4A protease. Hepatology.

[155] Choi J, Forman HJ, Ou JH, Lai MM, Seronello S, Nandipati A (2006). Redox modulation of the hepatitis C virus replication complex is calcium dependent. Free Radic Biol Med.

[156] Choi J, Lee KJ, Zheng Y, Yamaga AK, Lai MM, Ou JH (2004). Reactive oxygen species suppress hepatitis C virus RNA replication in human hepatoma cells. Hepatology.

[157] Kong L, Li S, Huang M, Xiong Y, Zhang Q, Ye L, Liu J, Zhu X, Sun R, Guo Y (2015). The roles of endoplasmic reticulum overload response induced by HCV and NS4B protein in human hepatocyte viability and virus replication. PLoS One.

[158] Seronello S, Montanez J, Presleigh K, Barlow M, Park SB, Choi J (2011). Ethanol and reactive species increase basal sequence heterogeneity of hepatitis C virus and produce variants with reduced susceptibility to antivirals. PLoS One.

[159] Forns X, Purcell RH, Bukh J (1999). Quasispecies in viral persistence and pathogenesis of hepatitis C virus. Trends Microbiol.

[160] Martinez C, Garcia-Martin E, Ladero JM, Herraez O, Ortega L, Taxonera C, Suarez A, Diaz-Rubio M, Agundez JA (2007). GSTT1 and GSTM1 null genotypes may facilitate hepatitis C virus infection becoming chronic. J Infect Dis.

[161] Harris C, Herker E, Farese RV, Ott M (2011). Hepatitis C virus core protein decreases lipid droplet turnover: a mechanism for core-induced steatosis. J Biol Chem.

[162] Boulant S, Vanbelle C, Ebel C, Penin F, Lavergne JP (2005). Hepatitis C virus core protein is a dimeric alpha-helical protein exhibiting membrane protein features. J Virol.

[163] Barba G, Harper F, Harada T, Kohara M, Goulinet S, Matsuura Y, Eder G, Schaff Z, Chapman MJ, Miyamura T, Brechot C (1997). Hepatitis C virus core protein shows a cytoplasmic localization and associates to cellular lipid storage droplets. Proc Natl Acad Sci U S A.

[164] Hope RG, McLauchlan J (2000). Sequence motifs required for lipid droplet association and protein stability are unique to the hepatitis C virus core protein. J Gen Virol.

[165] Mazumder N, Lyn RK, Singaravelu R, Ridsdale A, Moffatt DJ, Hu CW, Tsai HR, McLauchlan J, Stolow A, Kao FJ, Pezacki JP (2013). Fluorescence lifetime imaging of alterations to cellular metabolism by domain 2 of the hepatitis C virus core protein. PLoS One.

[166] Boulant S, Targett-Adams P, McLauchlan J (2007). Disrupting the association of hepatitis C virus core protein with lipid droplets correlates with a loss in production of infectious virus. J Gen Virol.

[167] Brault C, Levy PL, Bartosch B (2013). Hepatitis C virus-induced mitochondrial dysfunctions. Viruses.

[168] Lee J, Homma T, Kurahashi T, Kang ES, Fujii J (2015). Oxidative stress triggers lipid droplet accumulation in primary cultured hepatocytes by activating fatty acid synthesis. Biochem Biophys Res Commun.

[169] Lee SJ, Zhang J, Choi AM, Kim HP (2013). Mitochondrial dysfunction induces formation of lipid droplets as a generalized response to stress. Oxid Med Cell Longev.

[170] Imai H, Nakagawa Y (2003). Biological significance of phospholipid hydroperoxide glutathione peroxidase (PHGPx, GPx4) in mammalian cells. Free Radic Biol Med.

[171] Saeed M, Andreo U, Chung HY, Espiritu C, Branch AD, Silva JM, Rice CM (2015). SEC14L2 enables pan-genotype HCV replication in cell culture. Nature.

[172] Jack SC, Chan SW (2011). The role of PERK and GCN2 in basal and hydrogen peroxide-regulated translation from the hepatitis C virus internal ribosome entry site. Virus Genes.

[173] MacCallum PR, Jack SC, Egan PA, McDermott BT, Elliott RM, Chan SW (2006). Cap-dependent and hepatitis C virus internal ribosome entry site-mediated translation are modulated by phosphorylation of eIF2alpha under oxidative stress. J Gen Virol.

[174] Cao SS, Kaufman RJ (2012). Unfolded protein response. Curr Biol.

[175] Boltjes A, Movita D, Boonstra A, Woltman AM (2014). The role of Kupffer cells in hepatitis B and hepatitis C virus infections. J Hepatol.

[176] Canbay A, Feldstein AE, Higuchi H, Werneburg N, Grambihler A, Bronk SF, Gores GJ (2003). Kupffer cell engulfment of apoptotic bodies stimulates death ligand and cytokine expression. Hepatology.

[177] Chen W, Xu Y, Li H, Tao W, Xiang Y, Huang B, Niu J, Zhong J, Meng G (2014). HCV genomic RNA activates the NLRP3 inflammasome in human myeloid cells. PLoS One.

[178] Presser LD, Haskett A, Waris G (2011). Hepatitis C virus-induced furin and thrombospondin-1 activate TGF-beta1: role of TGF-beta1 in HCV replication. Virology.

[179] Jaeschke H (2000). Reactive oxygen and mechanisms of inflammatory liver injury. J Gastroenterol Hepatol.

[180] Lin W, Tsai WL, Shao RX, Wu G, Peng LF, Barlow LL, Chung WJ, Zhang L, Zhao H, Jang JY, Chung RT (2010). Hepatitis C virus regulates transforming growth factor beta1 production through the generation of reactive oxygen species in a nuclear factor kappaB-dependent manner. Gastroenterology.

[181] Bataller R, Lemon SM (2012). Fueling fibrosis in chronic hepatitis C. Proc Natl Acad Sci U S A.

[182] Kaimori A, Potter JJ, Choti M, Ding Z, Mezey E, Koteish AA (2010). Histone deacetylase inhibition suppresses the transforming growth factor beta1-induced epithelial-to-mesenchymal transition in hepatocytes. Hepatology.

[183] Xie G, Diehl AM (2013). Evidence for and against epithelial-to-mesenchymal transition in the liver. Am J Physiol Gastrointest Liver Physiol.

[184] Zeisberg M, Yang C, Martino M, Duncan MB, Rieder F, Tanjore H, Kalluri R (2007). Fibroblasts derive from hepatocytes in liver fibrosis via epithelial to mesenchymal transition. J Biol Chem.

[185] Friedman SL (2004). Mechanisms of disease: Mechanisms of hepatic fibrosis and therapeutic implications. Nat Clin Pract Gastroenterol Hepatol.

[186] Friedman SL (2008). Hepatic stellate cells: protean, multifunctional, and enigmatic cells of the liver. Physiol Rev.

[187] Ray S, Broor SL, Vaishnav Y, Sarkar C, Girish R, Dar L, Seth P, Broor S (2003). Transforming growth factor beta in hepatitis C virus infection: in vivo and in vitro findings. J Gastroenterol Hepatol.

[188] Tsushima H, Kawata S, Tamura S, Ito N, Shirai Y, Kiso S, Doi Y, Yamada A, Oshikawa O, Matsuzawa Y (1999). Reduced plasma transforming growth factor-beta1 levels in patients with chronic hepatitis C after interferon-alpha therapy: association with regression of hepatic fibrosis. J Hepatol.

[189] Bissell DM, Wang SS, Jarnagin WR, Roll FJ (1995). Cell-specific expression of transforming growth factor-beta in rat liver. Evidence for autocrine regulation of hepatocyte proliferation. J Clin Invest.

[190] Choi SS, Claridge LC, Jhaveri R, Swiderska-Syn M, Clark P, Suzuki A, Pereira TA, Mi Z, Kuo PC, Guy CD, Pereira FE, Diehl AM, Patel K (2014). Osteopontin is up-regulated in chronic hepatitis C and is associated with cellular permissiveness for hepatitis C virus replication. Clin Sci (Lond).

[191] Matsue Y, Tsutsumi M, Hayashi N, Saito T, Tsuchishima M, Toshikuni N, Arisawa T, George J (2015). Serum osteopontin predicts degree of hepatic fibrosis and serves as a biomarker in patients with hepatitis C virus infection. PLoS One.

[192] Huang W, Zhu G, Huang M, Lou G, Liu Y, Wang S (2010). Plasma osteopontin concentration correlates with the severity of hepatic fibrosis and inflammation in HCV-infected subjects. Clin Chim Acta.

[193] Iqbal J, McRae S, Banaudha K, Mai T, Waris G (2013). Mechanism of hepatitis C virus (HCV)-induced osteopontin and its role in epithelial to mesenchymal transition of hepatocytes. J Biol Chem.

[194] Urtasun R, Lopategi A, George J, Leung TM, Lu Y, Wang X, Ge X, Fiel MI, Nieto N (2012). Osteopontin, an oxidant stress sensitive cytokine, up-regulates collagen-I via integrin alpha(V)beta(3) engagement and PI3K/pAkt/NFkappaB signaling. Hepatology.

[195] Gieseler RK, Marquitan G, Schlattjan M, Sowa JP, Bechmann LP, Timm J, Roggendorf M, Gerken G, Friedman SL, Canbay A (2011). Hepatocyte apoptotic bodies encasing nonstructural HCV proteins amplify hepatic stellate cell activation: implications for chronic hepatitis C. J Viral Hepat.

[196] Wu CF, Lin YL, Huang YT (2012). Hepatitis C virus core protein stimulates fibrogenesis in hepatic stellate cells involving the obese receptor. J Cell Biochem.

[197] Nieto N, Friedman SL, Cederbaum AI (2002). Stimulation and proliferation of primary rat hepatic stellate cells by cytochrome P450 2E1-derived reactive oxygen species. Hepatology.

[198] Nanba S, Ikeda F, Baba N, Takaguchi K, Senoh T, Nagano T, Seki H, Takeuchi Y, Moritou Y, Yasunaka T, Ohnishi H, Miyake Y, Takaki A (2016). Association of hepatic oxidative stress and iron dysregulation with HCC development after interferon therapy in chronic hepatitis C. J Clin Pathol.

[199] Maki A, Kono H, Gupta M, Asakawa M, Suzuki T, Matsuda M, Fujii H, Rusyn I (2007). Predictive power of biomarkers of oxidative stress and inflammation in patients with hepatitis C virus-associated hepatocellular carcinoma. Ann Surg Oncol.

[200] Kryston TB, Georgiev AB, Pissis P, Georgakilas AG (2011). Role of oxidative stress and DNA damage in human carcinogenesis. Mutat Res.

[201] Chuma M, Hige S, Nakanishi M, Ogawa K, Natsuizaka M, Yamamoto Y, Asaka M (2008). 8-Hydroxy-2’-deoxy-guanosine is a risk factor for development of hepatocellular carcinoma in patients with chronic hepatitis C virus infection. J Gastroenterol Hepatol.

[202] Tamai T, Uto H, Takami Y, Oda K, Saishoji A, Hashiguchi M, Kumagai K, Kure T, Mawatari S, Moriuchi A, Oketani M, Ido A, Tsubouchi H (2011). Serum manganese superoxide dismutase and thioredoxin are potential prognostic markers for hepatitis C virus-related hepatocellular carcinoma. World J Gastroenterol.

[203] Tanaka H, Fujita N, Sugimoto R, Urawa N, Horiike S, Kobayashi Y, Iwasa M, Ma N, Kawanishi S, Watanabe S, Kaito M, Takei Y (2008). Hepatic oxidative DNA damage is associated with increased risk for hepatocellular carcinoma in chronic hepatitis C. Br J Cancer.

[204] Cardin R, Piciocchi M, Sinigaglia A, Lavezzo E, Bortolami M, Kotsafti A, Cillo U, Zanus G, Mescoli C, Rugge M, Farinati F (2012). Oxidative DNA damage correlates with cell immortalization and mir-92 expression in hepatocellular carcinoma. BMC Cancer.

[205] Piciocchi M, Cardin R, Cillo U, Vitale A, Cappon A, Mescoli C, Guido M, Rugge M, Burra P, Floreani A, Farinati F (2015). Differential timing of oxidative DNA damage and telomere shortening in hepatitis C and B virus-related liver carcinogenesis. Transl Res.

[206] Suzuki T, Harashima H, Kamiya H (2010). Effects of base excision repair proteins on mutagenesis by 8-oxo-7,8-dihydroguanine (8-hydroxyguanine) paired with cytosine and adenine. DNA Repair (Amst).

[207] Nishimura T, Kohara M, Izumi K, Kasama Y, Hirata Y, Huang Y, Shuda M, Mukaidani C, Takano T, Tokunaga Y, Nuriya H, Satoh M, Saito M (2009). Hepatitis C virus impairs p53 via persistent overexpression of 3beta-hydroxysterol Delta24-reductase. J Biol Chem.

[208] Tanaka N, Moriya K, Kiyosawa K, Koike K, Gonzalez FJ, Aoyama T (2008). PPARalpha activation is essential for HCV core protein-induced hepatic steatosis and hepatocellular carcinoma in mice. J Clin Invest.

[209] Mankouri J, Dallas ML, Hughes ME, Griffin SD, Macdonald A, Peers C, Harris M (2009). Suppression of a pro-apoptotic K+ channel as a mechanism for hepatitis C virus persistence. Proc Natl Acad Sci U S A.

[210] Seo YL, Heo S, Jang KL (2015). Hepatitis C virus core protein overcomes H2O2-induced apoptosis by downregulating p14 expression via DNA methylation. J Gen Virol.

[211] Kim SJ, Syed GH, Siddiqui A (2013). Hepatitis C virus induces the mitochondrial translocation of Parkin and subsequent mitophagy. PLoS Pathog.

[212] Dotto GP (2000). p21(WAF1/Cip1): more than a break to the cell cycle?. Biochim Biophys Acta.

[213] Gartel AL, Tyner AL (2002). The role of the cyclin-dependent kinase inhibitor p21 in apoptosis. Mol Cancer Ther.

[214] O’Reilly MA (2005). Redox activation of p21Cip1/WAF1/Sdi1: a multifunctional regulator of cell survival and death. Antioxid Redox Signal.

[215] Chen W, Sun Z, Wang XJ, Jiang T, Huang Z, Fang D, Zhang DD (2009). Direct interaction between Nrf2 and p21(Cip1/WAF1) upregulates the Nrf2-mediated antioxidant response. Mol Cell.

[216] Toledano MB (2009). The guardian recruits cops: the p53-p21 axis delegates prosurvival duties to the Keap1-Nrf2 stress pathway. Mol Cell.

[217] Villeneuve NF, Sun Z, Chen W, Zhang DD (2009). Nrf2 and p21 regulate the fine balance between life and death by controlling ROS levels. Cell Cycle.

[218] Fan S, Chang JK, Smith ML, Duba D, Fornace AJ, O’Connor PM (1997). Cells lacking CIP1/WAF1 genes exhibit preferential sensitivity to cisplatin and nitrogen mustard. Oncogene.

[219] Inoue T, Kato K, Kato H, Asanoma K, Kuboyama A, Ueoka Y, Yamaguchi S, Ohgami T, Wake N (2009). Level of reactive oxygen species induced by p21Waf1/CIP1 is critical for the determination of cell fate. Cancer Sci.

[220] Wang F, Yoshida I, Takamatsu M, Ishido S, Fujita T, Oka K, Hotta H (2000). Complex formation between hepatitis C virus core protein and p21Waf1/Cip1/Sdi1. Biochem Biophys Res Commun.

[221] Xiong Y, Hannon GJ, Zhang H, Casso D, Kobayashi R, Beach D (1993). p21 is a universal inhibitor of cyclin kinases. Nature.

[222] Marusawa H, Hijikata M, Chiba T, Shimotohno K (1999). Hepatitis C virus core protein inhibits Fas- and tumor necrosis factor alpha-mediated apoptosis via NF-kappaB activation. J Virol.

[223] Majumder M, Ghosh AK, Steele R, Ray R, Ray RB (2001). Hepatitis C virus NS5A physically associates with p53 and regulates p21/waf1 gene expression in a p53-dependent manner. J Virol.

[224] Hui AM, Kanai Y, Sakamoto M, Tsuda H, Hirohashi S (1997). Reduced p21(WAF1/CIP1) expression and p53 mutation in hepatocellular carcinomas. Hepatology.

[225] Kobayashi S, Matsushita K, Saigo K, Urashima T, Asano T, Hayashi H, Ochiai T (2001). P21WAF1/CIP1 messenger RNA expression in hepatitis B, C virus-infected human hepatocellular carcinoma tissues. Cancer.

[226] Kao JT, Chuah SK, Huang CC, Chen CL, Wang CC, Hung CH, Chen CH, Wang JH, Lu SN, Lee CM, Changchien CS, Hu TH (2007). P21/WAF1 is an independent survival prognostic factor for patients with hepatocellular carcinoma after resection. Liver Int.

[227] Wagayama H, Shiraki K, Sugimoto K, Ito T, Fujikawa K, Yamanaka T, Takase K, Nakano T (2002). High expression of p21WAF1/CIP1 is correlated with human hepatocellular carcinoma in patients with hepatitis C virus-associated chronic liver diseases. Hum Pathol.

[228] Haybaeck J, Zeller N, Wolf MJ, Weber A, Wagner U, Kurrer MO, Bremer J, Iezzi G, Graf R, Clavien PA, Thimme R, Blum H, Nedospasov SA (2009). A lymphotoxin-driven pathway to hepatocellular carcinoma. Cancer Cell.

[229] Simonin Y, Vegna S, Akkari L, Gregoire D, Antoine E, Piette J, Floc’h N, Lassus P, Yu GY, Rosenberg AR, Karin M, Durantel D, Hibner U (2013). Lymphotoxin signaling is initiated by the viral polymerase in HCV-linked tumorigenesis. PLoS Pathog.

[230] Lewis AK, Sachs JN (2014). Oxidative Stress Modulates Bioactivity of Lymphotoxin, but not TNF, Through Site-Specific Oxidation of Methionine Residues. Biophysical Journal.

[231] Wong GH, Kaspar RL, Zweiger G, Carlson C, Fong SE, Ehsani N, Vehar G (1996). Strategies for manipulating apoptosis for cancer therapy with tumor necrosis factor and lymphotoxin. J Cell Biochem.

[232] Moriya K, Nakagawa K, Santa T, Shintani Y, Fujie H, Miyoshi H, Tsutsumi T, Miyazawa T, Ishibashi K, Horie T, Imai K, Todoroki T, Kimura S (2001). Oxidative stress in the absence of inflammation in a mouse model for hepatitis C virus-associated hepatocarcinogenesis. Cancer Res.

[233] Ripoli M, D’Aprile A, Quarato G, Sarasin-Filipowicz M, Gouttenoire J, Scrima R, Cela O, Boffoli D, Heim MH, Moradpour D, Capitanio N, Piccoli C (2010). Hepatitis C virus-linked mitochondrial dysfunction promotes hypoxia-inducible factor 1 alpha-mediated glycolytic adaptation. J Virol.

[234] Levy PL, Duponchel S, Eischeid H, Molle J, Michelet M, Diserens G, Vermathen M, Vermathen P, Dufour JF, Dienes HP, Steffen HM, Odenthal M, Zoulim F (2016). Hepatitis C virus infection triggers a tumor-like glutamine metabolism. Hepatology.

[235] Hayes JD, Dinkova-Kostova AT (2014). The Nrf2 regulatory network provides an interface between redox and intermediary metabolism. Trends Biochem Sci.

[236] Miller DM, Thomas SD, Islam A, Muench D, Sedoris K (2012). c-Myc and cancer metabolism. Clin Cancer Res.

[237] Gordan JD, Thompson CB, Simon MC (2007). HIF and c-Myc: sibling rivals for control of cancer cell metabolism and proliferation. Cancer Cell.

[238] Chen JQ, Russo J (2012). Dysregulation of glucose transport, glycolysis, TCA cycle and glutaminolysis by oncogenes and tumor suppressors in cancer cells. Biochim Biophys Acta.

[239] Fosslien E (2001). Review: molecular pathology of cyclooxygenase-2 in cancer-induced angiogenesis. Ann Clin Lab Sci.

[240] Chang SH, Liu CH, Conway R, Han DK, Nithipatikom K, Trifan OC, Lane TF, Hla T (2004). Role of prostaglandin E2-dependent angiogenic switch in cyclooxygenase 2-induced breast cancer progression. Proc Natl Acad Sci U S A.

[241] (2015). Hepatitis B FAQs for the Public. (February 08: Centers for Disease Control and Prevention).

[242] Chang MH (2007). Hepatitis B virus infection. Semin Fetal Neonatal Med.

[243] 2015 Hepatitis B. Fact sheet N°204.

[244] Iannacone M, Sitia G, Ruggeri ZM, Guidotti LG (2007). HBV pathogenesis in animal models: recent advances on the role of platelets. J Hepatol.

[245] Fujita N, Sugimoto R, Ma N, Tanaka H, Iwasa M, Kobayashi Y, Kawanishi S, Watanabe S, Kaito M, Takei Y (2008). Comparison of hepatic oxidative DNA damage in patients with chronic hepatitis B and C. J Viral Hepat.

[246] Yuan K, Lei Y, Chen HN, Chen Y, Zhang T, Li K, Xie N, Wang K, Feng X, Pu Q, Yang W, Wu M, Xiang R (2016). HBV-induced ROS accumulation promotes hepatocarcinogenesis through Snail-mediated epigenetic silencing of SOCS3. Cell Death Differ.

[247] Duygu F, Karsen H, Aksoy N, Taskin A (2012). Relationship of oxidative stress in hepatitis B infection activity with HBV DNA and fibrosis. Ann Lab Med.

[248] Bolukbas C, Bolukbas FF, Horoz M, Aslan M, Celik H, Erel O (2005). Increased oxidative stress associated with the severity of the liver disease in various forms of hepatitis B virus infection. BMC Infect Dis.

[249] Bhargava A, Khan S, Panwar H, Pathak N, Punde RP, Varshney S, Mishra PK (2010). Occult hepatitis B virus infection with low viremia induces DNA damage, apoptosis and oxidative stress in peripheral blood lymphocytes. Virus Res.

[250] Dikici I, Mehmetoglu I, Dikici N, Bitirgen M, Kurban S (2005). Investigation of oxidative stress and some antioxidants in patients with acute and chronic viral hepatitis B and the effect of interferon-alpha treatment. Clin Biochem.

[251] Zhao J, Fan YC, Sun FK, Zhao ZH, Wang LY, Hu LH, Yin YP, Li T, Gao S, Wang K (2013). Peripheral type I interferon receptor correlated with oxidative stress in chronic hepatitis B virus infection. J Interferon Cytokine Res.

[252] Demirdag K, Yilmaz S, Ozdarendeli A, Ozden M, Kalkan A, Kilic SS (2003). Levels of plasma malondialdehyde and erythrocyte antioxidant enzyme activities in patients with chronic hepatitis B. Hepatogastroenterology.

[253] Tasdelen Fisgin N, Aydin BK, Sarikaya H, Tanyel E, Esen S, Sunbul M, Leblebicioglu H (2012). Oxidative stress and antioxidant defense in patients with chronic hepatitis B. Clin Lab.

[254] Popadiuk S, Liberek A, Korzon M, Renke J, Wozniak M (2004). Free radical reactions and activity of antioxidant barrier in children with chronic hepatitis B. Med Wieku Rozwoj.

[255] Ishizakai M, Yoshida K, Nishimoto N, Saleh AM, Ishii C, Handa H, Ogawara H, Nagamine T, Murakami M, Murakami H (2004). Urinary 8-hydroxy-2’-deoxyguanosin (8-OHdG) in patients with chronic liver diseases. Rinsho Byori.

[256] Qi L, Zou ZQ, Wang LY, Gao S, Fan YC, Long B, Guo YM, Xu AL, Han J, Li T, Wang K (2012). Methylation of the glutathione-S-transferase M3 gene promoter is associated with oxidative stress in acute-on-chronic hepatitis B liver failure. Tohoku J Exp Med.

[257] Cai J, Han T, Nie C, Jia X, Liu Y, Zhu Z, Gao Y (2015). Biomarkers of oxidation stress, inflammation, necrosis and apoptosis are associated with hepatitis B-related acute-on-chronic liver failure. Clin Res Hepatol Gastroenterol.

[258] Liu H, Han T, Tian J, Zhu ZY, Liu Y, Li Y, Xiao SX, Feng YY (2012). Monitoring oxidative stress in acute-on-chronic liver failure by advanced oxidation protein products. Hepatol Res.

[259] Lee YI, Hwang JM, Im JH, Kim NS, Kim DG, Yu DY, Moon HB, Park SK (2004). Human hepatitis B virus-X protein alters mitochondrial function and physiology in human liver cells. J Biol Chem.

[260] Waris G, Huh KW, Siddiqui A (2001). Mitochondrially associated hepatitis B virus X protein constitutively activates transcription factors STAT-3 and NF-kappa B via oxidative stress. Mol Cell Biol.

[261] Jung SY, Kim YJ (2013). C-terminal region of HBx is crucial for mitochondrial DNA damage. Cancer Lett.

[262] Ren JH, Chen X, Zhou L, Tao NN, Zhou HZ, Liu B, Li WY, Huang AL, Chen J (2016). Protective Role of Sirtuin3 (SIRT3) in Oxidative Stress Mediated by Hepatitis B Virus X Protein Expression. PLoS One.

[263] Kim H, Lee SA, Won YS, Lee H, Kim BJ (2015). Occult infection related hepatitis B surface antigen variants showing lowered secretion capacity. World J Gastroenterol.

[264] Lee IK, Lee SA, Kim H, Won YS, Kim BJ (2015). Induction of endoplasmic reticulum-derived oxidative stress by an occult infection related S surface antigen variant. World J Gastroenterol.

[265] Lee H, Kim H, Lee SA, Won YS, Kim HI, Inn KS, Kim BJ (2015). Upregulation of endoplasmic reticulum stress and reactive oxygen species by naturally occurring mutations in hepatitis B virus core antigen. J Gen Virol.

[266] Lim W, Kwon SH, Cho H, Kim S, Lee S, Ryu WS (2010). HBx targeting to mitochondria and ROS generation are necessary but insufficient for HBV-induced cyclooxygenase-2 expression. J Mol Med (Berl).

[267] Henkler F, Hoare J, Waseem N, Goldin RD, McGarvey MJ, Koshy R, King IA (2001). Intracellular localization of the hepatitis B virus HBx protein. J Gen Virol.

[268] McClain SL, Clippinger AJ, Lizzano R, Bouchard MJ (2007). Hepatitis B virus replication is associated with an HBx-dependent mitochondrion-regulated increase in cytosolic calcium levels. J Virol.

[269] Shirakata Y, Koike K (2003). Hepatitis B virus X protein induces cell death by causing loss of mitochondrial membrane potential. J Biol Chem.

[270] Li SK, Ho SF, Tsui KW, Fung KP, Waye MY (2008). Identification of functionally important amino acid residues in the mitochondria targeting sequence of hepatitis B virus X protein. Virology.

[271] Rahmani Z, Huh KW, Lasher R, Siddiqui A (2000). Hepatitis B virus X protein colocalizes to mitochondria with a human voltage-dependent anion channel, HVDAC3, and alters its transmembrane potential. J Virol.

[272] Takada S, Shirakata Y, Kaneniwa N, Koike K (1999). Association of hepatitis B virus X protein with mitochondria causes mitochondrial aggregation at the nuclear periphery, leading to cell death. Oncogene.

[273] Zheng BY, Fang XF, Zou LY, Huang YH, Chen ZX, Li D, Zhou LY, Chen H, Wang XZ (2014). The co-localization of HBx and COXIII upregulates COX-2 promoting HepG2 cell growth. Int J Oncol.

[274] Zou LY, Zheng BY, Fang XF, Li D, Huang YH, Chen ZX, Zhou LY, Wang XZ (2015). HBx co-localizes with COXIII in HL-7702 cells to upregulate mitochondrial function and ROS generation. Oncol Rep.

[275] Clippinger AJ, Bouchard MJ (2008). Hepatitis B virus HBx protein localizes to mitochondria in primary rat hepatocytes and modulates mitochondrial membrane potential. J Virol.

[276] Yang B, Bouchard MJ (2012). The hepatitis B virus X protein elevates cytosolic calcium signals by modulating mitochondrial calcium uptake. J Virol.

[277] De Stefani D, Bononi A, Romagnoli A, Messina A, De Pinto V, Pinton P, Rizzuto R (2012). VDAC1 selectively transfers apoptotic Ca2+ signals to mitochondria. Cell Death Differ.

[278] Li D, Wang XZ, Yu JP, Chen ZX, Huang YH, Tao QM (2004). Cytochrome C oxidase III interacts with hepatitis B virus X protein in vivo by yeast two-hybrid system. World J Gastroenterol.

[279] Kadenbach B, Huttemann M (2015). The subunit composition and function of mammalian cytochrome c oxidase. Mitochondrion.

[280] Li D, Ding J, Chen Z, Chen Y, Lin N, Chen F, Wang X (2015). Accurately mapping the location of the binding site for the interaction between hepatitis B virus X protein and cytochrome c oxidase III. Int J Mol Med.

[281] Hsieh YH, Su IJ, Wang HC, Chang WW, Lei HY, Lai MD, Chang WT, Huang W (2004). Pre-S mutant surface antigens in chronic hepatitis B virus infection induce oxidative stress and DNA damage. Carcinogenesis.

[282] Gerlich WH (2013). Medical virology of hepatitis B: how it began and where we are now. Virol J.

[283] Peiffer KH, Akhras S, Himmelsbach K, Hassemer M, Finkernagel M, Carra G, Nuebling M, Chudy M, Niekamp H, Glebe D, Sarrazin C, Zeuzem S, Hildt E (2015). Intracellular accumulation of subviral HBsAg particles and diminished Nrf2 activation in HBV genotype G expressing cells lead to an increased ROI level. J Hepatol.

[284] Gwak GY, Lee DH, Moon TG, Choi MS, Lee JH, Koh KC, Paik SW, Park CK, Joh JW, Yoo BC (2008). The correlation of hepatitis B virus pre-S mutation with cellular oxidative DNA damage in hepatocellular carcinoma. Hepatogastroenterology.

[285] Schluter V, Rabe C, Meyer M, Koshy R, Caselmann WH (2001). Intracellular accumulation of middle hepatitis B surface protein activates gene transcription. Dig Dis.

[286] Kaufman RJ (1999). Stress signaling from the lumen of the endoplasmic reticulum: coordination of gene transcriptional and translational controls. Genes Dev.

[287] Schaedler S, Krause J, Himmelsbach K, Carvajal-Yepes M, Lieder F, Klingel K, Nassal M, Weiss TS, Werner S, Hildt E (2010). Hepatitis B virus induces expression of antioxidant response element-regulated genes by activation of Nrf2. J Biol Chem.

[288] Li H, Zhu W, Zhang L, Lei H, Wu X, Guo L, Chen X, Wang Y, Tang H (2015). The metabolic responses to hepatitis B virus infection shed new light on pathogenesis and targets for treatment. Sci Rep.

[289] Severi T, Ying C, Vermeesch JR, Cassiman D, Cnops L, Verslype C, Fevery J, Arckens L, Neyts J, van Pelt JF (2006). Hepatitis B virus replication causes oxidative stress in HepAD38 liver cells. Mol Cell Biochem.

[290] Ohtsuji M, Katsuoka F, Kobayashi A, Aburatani H, Hayes JD, Yamamoto M (2008). Nrf1 and Nrf2 play distinct roles in activation of antioxidant response element-dependent genes. J Biol Chem.

[291] Liu B, Fang M, He Z, Cui D, Jia S, Lin X, Xu X, Zhou T, Liu W (2015). Hepatitis B virus stimulates G6PD expression through HBx-mediated Nrf2 activation. Cell Death Dis.

[292] Wu YL, Wang D, Peng XE, Chen YL, Zheng DL, Chen WN, Lin X (2013). Epigenetic silencing of NAD(P)H:quinone oxidoreductase 1 by hepatitis B virus X protein increases mitochondrial injury and cellular susceptibility to oxidative stress in hepatoma cells. Free Radic Biol Med.

[293] Huang Q, Wang L, Bai S, Lin W, Chen W, Lin J, Lin X (2009). Global proteome analysis of hepatitis B virus expressing human hepatoblastoma cell line HepG2. J Med Virol.

[294] Niu D, Zhang J, Ren Y, Feng H, Chen WN (2009). HBx genotype D represses GSTP1 expression and increases the oxidative level and apoptosis in HepG2 cells. Mol Oncol.

[295] Tong A, Wu L, Lin Q, Lau QC, Zhao X, Li J, Chen P, Chen L, Tang H, Huang C, Wei YQ (2008). Proteomic analysis of cellular protein alterations using a hepatitis B virus-producing cellular model. Proteomics.

[296] Cho IJ, Ki SH, Brooks C, Kim SG (2009). Role of hepatitis B virus X repression of C/EBPbeta activity in the down-regulation of glutathione S-transferase A2 gene: implications in other phase II detoxifying enzyme expression. Xenobiotica.

[297] Li T, Meng QH, Zou ZQ, Fan YC, Long B, Guo YM, Hou W, Zhao J, Li J, Yu HW, Zhu YK, Wang K (2011). Correlation between promoter methylation of glutathione-S-tranferase P1 and oxidative stress in acute-on-chronic hepatitis B liver failure. J Viral Hepat.

[298] Ding C, Wei H, Sun R, Zhang J, Tian Z (2009). Hepatocytes proteomic alteration and seroproteome analysis of HBV-transgenic mice. Proteomics.

[299] Wu KC, Cui JY, Klaassen CD (2012). Effect of graded Nrf2 activation on phase-I and -II drug metabolizing enzymes and transporters in mouse liver. PLoS One.

[300] Yao D, Li H, Gou Y, Zhang H, Vlessidis AG, Zhou H, Evmiridis NP, Liu Z (2009). Betulinic acid-mediated inhibitory effect on hepatitis B virus by suppression of manganese superoxide dismutase expression. FEBS J.

[301] Wang Q, Na B, Ou JH, Pulliam L, Yen TS (2012). Hepatitis B virus alters the antioxidant system in transgenic mice and sensitizes hepatocytes to Fas signaling. PLoS One.

[302] Yi YS, Park SG, Byeon SM, Kwon YG, Jung G (2003). Hepatitis B virus X protein induces TNF-alpha expression via down-regulation of selenoprotein P in human hepatoma cell line, HepG2. Biochim Biophys Acta.

[303] Burk RF, Hill KE (2009). Selenoprotein P-expression, functions, and roles in mammals. Biochim Biophys Acta.

[304] Steinbrenner H, Sies H (2009). Protection against reactive oxygen species by selenoproteins. Biochim Biophys Acta.

[305] Petersen J, Dandri M, Burkle A, Zhang L, Rogler CE (1997). Increase in the frequency of hepadnavirus DNA integrations by oxidative DNA damage and inhibition of DNA repair. J Virol.

[306] Zheng YW, Yen TS (1994). Negative regulation of hepatitis B virus gene expression and replication by oxidative stress. J Biol Chem.

[307] Wang JH, Yun C, Kim S, Lee JH, Yoon G, Lee MO, Cho H (2003). Reactive oxygen species modulates the intracellular level of HBx viral oncoprotein. Biochem Biophys Res Commun.

[308] Yun C, Lee JH, Wang JH, Seong JK, Oh SH, Yu DY, Cho H (2002). Expression of hepatitis B virus X (HBx) gene is up-regulated by adriamycin at the post-transcriptional level. Biochem Biophys Res Commun.

[309] Min BY, Kim NY, Jang ES, Shin CM, Lee SH, Park YS, Hwang JH, Jeong SH, Kim N, Lee DH, Kim JW (2013). Ethanol potentiates hepatitis B virus replication through oxidative stress-dependent and -independent transcriptional activation. Biochem Biophys Res Commun.

[310] Kim YS, Seo HW, Jung G (2015). Reactive oxygen species promote heat shock protein 90-mediated HBV capsid assembly. Biochem Biophys Res Commun.

[311] Dandri M, Burda MR, Burkle A, Zuckerman DM, Will H, Rogler CE, Greten H, Petersen J (2002). Increase in de novo HBV DNA integrations in response to oxidative DNA damage or inhibition of poly(ADP-ribosyl)ation. Hepatology.

[312] Sen V, Uluca U, Ece A, Kaplan I, Bozkurt F, Aktar F, Bagli S, Tekin R (2014). Serum prolidase activity and oxidant-antioxidant status in children with chronic hepatitis B virus infection. Ital J Pediatr.

[313] Osna NA, White RL, Todero S, McVicker BL, Thiele GM, Clemens DL, Tuma DJ, Donohue TM (2007). Ethanol-induced oxidative stress suppresses generation of peptides for antigen presentation by hepatoma cells. Hepatology.

[314] Fan XP, Wang K, Liu Y, Wang JF (2009). Plasma alpha-tocopherol is negatively correlated with hepatocyte apoptosis in chronic hepatitis B patients. Intern Med.

[315] Su F, Schneider RJ (1996). Hepatitis B virus HBx protein activates transcription factor NF-kappaB by acting on multiple cytoplasmic inhibitors of rel-related proteins. J Virol.

[316] Lou X, Hou Y, Liang D (2013). Effects of hepatitis B virus X protein on human T cell cytokines. Can J Microbiol.

[317] Lara-Pezzi E, Majano PL, Gomez-Gonzalo M, Garcia-Monzon C, Moreno-Otero R, Levrero M, Lopez-Cabrera M (1998). The hepatitis B virus X protein up-regulates tumor necrosis factor alpha gene expression in hepatocytes. Hepatology.

[318] Lee SH, Park SG, Lim SO, Jung G (2005). The hepatitis B virus X protein up-regulates lymphotoxin alpha expression in hepatocytes. Biochim Biophys Acta.

[319] Chen WN, Liu LL, Jiao BY, Lin WS, Lin XJ, Lin X (2015). Hepatitis B virus X protein increases the IL-1beta-induced NF-kappaB activation via interaction with evolutionarily conserved signaling intermediate in Toll pathways (ECSIT). Virus Res.

[320] Xiang WQ, Feng WF, Ke W, Sun Z, Chen Z, Liu W (2011). Hepatitis B virus X protein stimulates IL-6 expression in hepatocytes via a MyD88-dependent pathway. J Hepatol.

[321] Xia LM, Huang WJ, Wu JG, Yang YB, Zhang Q, Zhou ZZ, Zhu HF, Lei P, Shen GX, Tian DA (2009). HBx protein induces expression of MIG and increases migration of leukocytes through activation of NF-kappaB. Virology.

[322] Zhou Y, Wang S, Ma JW, Lei Z, Zhu HF, Lei P, Yang ZS, Zhang B, Yao XX, Shi C, Sun LF, Wu XW, Ning Q (2010). Hepatitis B virus protein X-induced expression of the CXC chemokine IP-10 is mediated through activation of NF-kappaB and increases migration of leukocytes. J Biol Chem.

[323] Cho HK, Kim SY, Yoo SK, Choi YH, Cheong J (2014). Fatty acids increase hepatitis B virus X protein stabilization and HBx-induced inflammatory gene expression. FEBS J.

[324] Hinz B, Brune K (2002). Cyclooxygenase-2--10 years later. J Pharmacol Exp Ther.

[325] Kim SH, Oh JM, No JH, Bang YJ, Juhnn YS, Song YS (2009). Involvement of NF-kappaB and AP-1 in COX-2 upregulation by human papillomavirus 16 E5 oncoprotein. Carcinogenesis.

[326] Li T, Zhao XP, Wang LY, Gao S, Zhao J, Fan YC, Wang K (2013). Glutathione S-transferase P1 correlated with oxidative stress in hepatocellular carcinoma. Int J Med Sci.

[327] Gao S, Sun FK, Fan YC, Shi CH, Zhang ZH, Wang LY, Wang K (2015). Aberrant GSTP1 promoter methylation predicts short-term prognosis in acute-on-chronic hepatitis B liver failure. Aliment Pharmacol Ther.

[328] Herceg Z, Hainaut P (2007). Genetic and epigenetic alterations as biomarkers for cancer detection, diagnosis and prognosis. Mol Oncol.

[329] Woodson K, O’Reilly KJ, Hanson JC, Nelson D, Walk EL, Tangrea JA (2008). The usefulness of the detection of GSTP1 methylation in urine as a biomarker in the diagnosis of prostate cancer. J Urol.

[330] Hu L, Chen L, Yang G, Li L, Sun H, Chang Y, Tu Q, Wu M, Wang H (2011). HBx sensitizes cells to oxidative stress-induced apoptosis by accelerating the loss of Mcl-1 protein via caspase-3 cascade. Mol Cancer.

[331] Severi T, Vander Borght S, Libbrecht L, VanAelst L, Nevens F, Roskams T, Cassiman D, Fevery J, Verslype C, van Pelt JF (2007). HBx or HCV core gene expression in HepG2 human liver cells results in a survival benefit against oxidative stress with possible implications for HCC development. Chem Biol Interact.

[332] Kim YJ, Jung JK, Lee SY, Jang KL (2010). Hepatitis B virus X protein overcomes stress-induced premature senescence by repressing p16(INK4a) expression via DNA methylation. Cancer Lett.

[333] Srisuttee R, Koh SS, Park EH, Cho IR, Min HJ, Jhun BH, Yu DY, Park S, Park do Y, Lee MO, Castrillon DH, Johnston RN, Chung YH (2011). Up-regulation of Foxo4 mediated by hepatitis B virus X protein confers resistance to oxidative stress-induced cell death. Int J Mol Med.

[334] Srisuttee R, Koh SS, Malilas W, Moon J, Cho IR, Jhun BH, Horio Y, Chung YH (2012). SIRT1 sensitizes hepatocellular carcinoma cells expressing hepatitis B virus X protein to oxidative stress-induced apoptosis. Biochem Biophys Res Commun.

[335] Dayoub R, Vogel A, Schuett J, Lupke M, Spieker SM, Kettern N, Hildt E, Melter M, Weiss TS (2013). Nrf2 activates augmenter of liver regeneration (ALR) via antioxidant response element and links oxidative stress to liver regeneration. Mol Med.

[336] Burgering BM, Medema RH (2003). Decisions on life and death: FOXO Forkhead transcription factors are in command when PKB/Akt is off duty. J Leukoc Biol.

[337] Tsai SM, Lin SK, Lee KT, Hsiao JK, Huang JC, Wu SH, Ma H, Tsai LY (2009). Evaluation of redox statuses in patients with hepatitis B virus-associated hepatocellular carcinoma. Ann Clin Biochem.

[338] Sumiyoshi S, Kobayashi Y, Kawamura K, Kawata K, Nakamura H (2013). Differential expression of hepatic apurinic/apyrimidinic endonuclease 1, a DNA repair enzyme, in chronic hepatitis. World J Hepatol.

[339] Kim S, Lee HS, Ji JH, Cho MY, Yoo YS, Park YY, Cha HJ, Lee Y, Kim Y, Cho H (2015). Hepatitis B virus X protein activates the ATM-Chk2 pathway and delays cell cycle progression. J Gen Virol.

[340] Smith J, Tho LM, Xu N, Gillespie DA (2010). The ATM-Chk2 and ATR-Chk1 pathways in DNA damage signaling and cancer. Adv Cancer Res.

[341] Paterlini-Brechot P, Saigo K, Murakami Y, Chami M, Gozuacik D, Mugnier C, Lagorce D, Brechot C (2003). Hepatitis B virus-related insertional mutagenesis occurs frequently in human liver cancers and recurrently targets human telomerase gene. Oncogene.

[342] Toh ST, Jin Y, Liu L, Wang J, Babrzadeh F, Gharizadeh B, Ronaghi M, Toh HC, Chow PK, Chung AY, Ooi LL, Lee CG (2013). Deep sequencing of the hepatitis B virus in hepatocellular carcinoma patients reveals enriched integration events, structural alterations and sequence variations. Carcinogenesis.

[343] Sung WK, Zheng H, Li S, Chen R, Liu X, Li Y, Lee NP, Lee WH, Ariyaratne PN, Tennakoon C, Mulawadi FH, Wong KF, Liu AM (2012). Genome-wide survey of recurrent HBV integration in hepatocellular carcinoma. Nat Genet.

[344] Arbuthnot P, Kew M (2001). Hepatitis B virus and hepatocellular carcinoma. Int J Exp Pathol.

[345] Murakami Y, Saigo K, Takashima H, Minami M, Okanoue T, Brechot C, Paterlini-Brechot P (2005). Large scaled analysis of hepatitis B virus (HBV) DNA integration in HBV related hepatocellular carcinomas. Gut.

[346] Poungpairoj P, Whongsiri P, Suwannasin S, Khlaiphuengsin A, Tangkijvanich P, Boonla C (2015). Increased Oxidative Stress and RUNX3 Hypermethylation in Patients with Hepatitis B Virus-Associated Hepatocellular Carcinoma (HCC) and Induction of RUNX3 Hypermethylation by Reactive Oxygen Species in HCC Cells. Asian Pac J Cancer Prev.

[347] Chuang LS, Ito Y (2010). RUNX3 is multifunctional in carcinogenesis of multiple solid tumors. Oncogene.

[348] Chen J, Siddiqui A (2007). Hepatitis B virus X protein stimulates the mitochondrial translocation of Raf-1 via oxidative stress. J Virol.

[349] Matallanas D, Birtwistle M, Romano D, Zebisch A, Rauch J, von Kriegsheim A, Kolch W (2011). Raf family kinases: old dogs have learned new tricks. Genes Cancer.

[350] Ha HL, Yu DY (2010). HBx-induced reactive oxygen species activates hepatocellular carcinogenesis via dysregulation of PTEN/Akt pathway. World J Gastroenterol.

[351] Fresno Vara JA, Casado E, de Castro J, Cejas P, Belda-Iniesta C, Gonzalez-Baron M (2004). PI3K/Akt signalling pathway and cancer. Cancer Treat Rev.

[352] Wang MD, Wu H, Huang S, Zhang HL, Qin CJ, Zhao LH, Fu GB, Zhou X, Wang XM, Tang L, Wen W, Yang W, Tang SH (2016). HBx regulates fatty acid oxidation to promote hepatocellular carcinoma survival during metabolic stress. Oncotarget.

[353] Teng CF, Hsieh WC, Yang CW, Su HM, Tsai TF, Sung WC, Huang W, Su IJ (2016). A biphasic response pattern of lipid metabolomics in the stage progression of hepatitis B virus X tumorigenesis. Mol Carcinog.

[354] Na TY, Shin YK, Roh KJ, Kang SA, Hong I, Oh SJ, Seong JK, Park CK, Choi YL, Lee MO (2009). Liver X receptor mediates hepatitis B virus X protein-induced lipogenesis in hepatitis B virus-associated hepatocellular carcinoma. Hepatology.

[355] Kim KH, Shin HJ, Kim K, Choi HM, Rhee SH, Moon HB, Kim HH, Yang US, Yu DY, Cheong J (2007). Hepatitis B virus X protein induces hepatic steatosis via transcriptional activation of SREBP1 and PPARgamma. Gastroenterology.

[356] Videla LA, Pettinelli P (2012). Misregulation of PPAR Functioning and Its Pathogenic Consequences Associated with Nonalcoholic Fatty Liver Disease in Human Obesity. PPAR Res.

[357] Waris G, Felmlee DJ, Negro F, Siddiqui A (2007). Hepatitis C virus induces proteolytic cleavage of sterol regulatory element binding proteins and stimulates their phosphorylation via oxidative stress. J Virol.

[358] Bose S, Tripathi DM, Sukriti, Sakhuja P, Kazim SN, Sarin SK (2013). Genetic polymorphisms of CYP2E1 and DNA repair genes HOGG1 and XRCC1: association with hepatitis B related advanced liver disease and cancer. Gene.

[359] Liu Y, Xie L, Zhao J, Huang X, Song L, Luo J, Ma L, Li S, Qin X (2015). Association between catalase gene polymorphisms and risk of chronic hepatitis B, hepatitis B virus-related liver cirrhosis and hepatocellular carcinoma in Guangxi population: a case-control study. Medicine (Baltimore).

[360] Shim JJ, Oh IH, Kim SB, Kim JW, Lee CK, Jang JY, Lee JS, Kim BH (2016). Predictive Value of Antiviral Effects in the Development of Hepatocellular Carcinoma in the General Korean Population with Chronic Hepatitis B. Gut Liver.

[361] Wang JP, Kao FY, Wu CY, Hung YP, Chao Y, Chou YJ, Li CP (2015). Nucleos(t)ide analogues associated with a reduced risk of hepatocellular carcinoma in hepatitis B patients: a population-based cohort study. Cancer.

[362] Coffin CS, Rezaeeaval M, Pang JX, Alcantara L, Klein P, Burak KW, Myers RP (2014). The incidence of hepatocellular carcinoma is reduced in patients with chronic hepatitis B on long-term nucleos(t)ide analogue therapy. Aliment Pharmacol Ther.

[363] Wu CY, Lin JT, Ho HJ, Su CW, Lee TY, Wang SY, Wu C, Wu JC (2014). Association of nucleos(t)ide analogue therapy with reduced risk of hepatocellular carcinoma in patients with chronic hepatitis B: a nationwide cohort study. Gastroenterology.

[364] Hoang JK, Yang HI, Le A, Nguyen NH, Lin D, Vu VD, Chaung K, Nguyen V, Trinh HN, Li J, Zhang JQ, Chen CJ, Nguyen MH (2016). Lower liver cancer risk with antiviral therapy in chronic hepatitis B patients with normal to minimally elevated ALT and no cirrhosis. Medicine (Baltimore).

[365] Su TH, Hu TH, Chen CY, Huang YH, Chuang WL, Lin CC, Wang CC, Su WW, Chen MY, Peng CY, Chien RN, Huang YW, Wang HY (2016). Four-year entecavir therapy reduces hepatocellular carcinoma, cirrhotic events and mortality in chronic hepatitis B patients. Liver Int.

[366] Sinn DH, Lee J, Goo J, Kim K, Gwak GY, Paik YH, Choi MS, Lee JH, Koh KC, Yoo BC, Paik SW (2015). Hepatocellular carcinoma risk in chronic hepatitis B virus-infected compensated cirrhosis patients with low viral load. Hepatology.

[367] Kim SS, Hwang JC, Lim SG, Ahn SJ, Cheong JY, Cho SW (2014). Effect of virological response to entecavir on the development of hepatocellular carcinoma in hepatitis B viral cirrhotic patients: comparison between compensated and decompensated cirrhosis. Am J Gastroenterol.

[368] Kim JH, Sinn DH, Kim K, Kim H, Gwak GY, Paik YH, Choi MS, Lee JH, Koh KC, Paik SW (2016). Lamivudine versus Entecavir for Newly Diagnosed Hepatitis B Virus-Related Hepatocellular Carcinoma. Gut Liver.

[369] Tsai MC, Chen CH, Hu TH, Lu SN, Lee CM, Wang JH, Hung CH (2016). Long-term outcomes of hepatitis B virus-related cirrhosis treated with nucleos(t)ide analogs. J Formos Med Assoc.

[370] Lim YS, Han S, Heo NY, Shim JH, Lee HC, Suh DJ (2014). Mortality, liver transplantation, and hepatocellular carcinoma among patients with chronic hepatitis B treated with entecavir vs lamivudine. Gastroenterology.

[371] Liang KH, Hsu CW, Chang ML, Chen YC, Lai MW, Yeh CT (2016). Peginterferon Is Superior to Nucleos(t)ide Analogues for Prevention of Hepatocellular Carcinoma in Chronic Hepatitis B. J Infect Dis.

[372] Arends P, Sonneveld MJ, Zoutendijk R, Carey I, Brown A, Fasano M, Mutimer D, Deterding K, Reijnders JG, Oo Y, Petersen J, van Bommel F, de Knegt RJ (2015). Entecavir treatment does not eliminate the risk of hepatocellular carcinoma in chronic hepatitis B: limited role for risk scores in Caucasians. Gut.

[373] Cho JY, Paik YH, Sohn W, Cho HC, Gwak GY, Choi MS, Lee JH, Koh KC, Paik SW, Yoo BC (2014). Patients with chronic hepatitis B treated with oral antiviral therapy retain a higher risk for HCC compared with patients with inactive stage disease. Gut.

[374] Li Z, Zhao X, Jiang P, Xiao S, Wu G, Chen K, Zhang X, Liu H, Han X, Wang S, Li X (2016). HBV is a risk factor for poor patient prognosis after curative resection of hepatocellular carcinoma: A retrospective case-control study. Medicine (Baltimore).

[375] Wong GL, Tse YK, Chan HL, Yip TC, Tsoi KK, Wong VW (2016). Oral nucleos(t)ide analogues reduce recurrence and death in chronic hepatitis B-related hepatocellular carcinoma. Aliment Pharmacol Ther.

[376] Lee TY, Lin JT, Zeng YS, Chen YJ, Wu MS, Wu CY (2016). Association between nucleos(t)ide analog and tumor recurrence in hepatitis B virus-related hepatocellular carcinoma after radiofrequency ablation. Hepatology.

[377] Lee YA, Friedman SL (2014). Reversal, maintenance or progression: what happens to the liver after a virologic cure of hepatitis C?. Antiviral Res.

[378] John-Baptiste AA, Tomlinson G, Hsu PC, Krajden M, Heathcote EJ, Laporte A, Yoshida EM, Anderson FH, Krahn MD (2009). Sustained responders have better quality of life and productivity compared with treatment failures long after antiviral therapy for hepatitis C. Am J Gastroenterol.

[379] Belli LS, Berenguer M, Cortesi PA, Strazzabosco M, Rockenschaub SR, Martini S, Morelli C, Donato F, Volpes R, Pageaux GP, Coilly A, Fagiuoli S, Amaddeo G (2016). Delisting of liver transplant candidates with chronic hepatitis C after viral eradication: A European study. J Hepatol.

[380] Poynard T, Moussalli J, Munteanu M, Thabut D, Lebray P, Rudler M, Ngo Y, Thibault V, Mkada H, Charlotte F, Bismut FI, Deckmyn O, Benhamou Y (2013). Slow regression of liver fibrosis presumed by repeated biomarkers after virological cure in patients with chronic hepatitis C. J Hepatol.

[381] Maylin S, Martinot-Peignoux M, Moucari R, Boyer N, Ripault MP, Cazals-Hatem D, Giuily N, Castelnau C, Cardoso AC, Asselah T, Feray C, Nicolas-Chanoine MH, Bedossa P (2008). Eradication of hepatitis C virus in patients successfully treated for chronic hepatitis C. Gastroenterology.

[382] D’Ambrosio R, Aghemo A, Rumi MG, Ronchi G, Donato MF, Paradis V, Colombo M, Bedossa P (2012). A morphometric and immunohistochemical study to assess the benefit of a sustained virological response in hepatitis C virus patients with cirrhosis. Hepatology.

[383] Nahon P, Bourcier V, Layese R, Audureau E, Cagnot C, Marcellin P, Guyader D, Fontaine H, Larrey D, De Ledinghen V, Ouzan D, Zoulim F, Roulot D (2016). Eradication of Hepatitis C Virus Infection in Patients With Cirrhosis Reduces Risk of Liver and Non-Liver Complications. Gastroenterology.

[384] Cheung MC, Walker AJ, Hudson BE, Verma S, McLauchlan J, Mutimer DJ, Brown A, Gelson WT, MacDonald DC, Agarwal K, Foster GR, Irving WL (2016). Outcomes after successful direct-acting antiviral therapy for patients with chronic hepatitis C and decompensated cirrhosis. J Hepatol.

[385] Akuta N, Suzuki F, Seko Y, Kawamura Y, Sezaki H, Suzuki Y, Hosaka T, Kobayashi M, Saitoh S, Arase Y, Ikeda K, Kumada H (2013). Efficacy and anticarcinogenic activity of ribavirin combination therapy for hepatitis C virus-related compensated cirrhosis. Intervirology.

[386] Aleman S, Rahbin N, Weiland O, Davidsdottir L, Hedenstierna M, Rose N, Verbaan H, Stal P, Carlsson T, Norrgren H, Ekbom A, Granath F, Hultcrantz R (2013). A risk for hepatocellular carcinoma persists long-term after sustained virologic response in patients with hepatitis C-associated liver cirrhosis. Clin Infect Dis.

[387] Conti F, Buonfiglioli F, Scuteri A, Crespi C, Bolondi L, Caraceni P, Foschi FG, Lenzi M, Mazzella G, Verucchi G, Andreone P, Brillanti S (2016). Early occurrence and recurrence of hepatocellular carcinoma in HCV-related cirrhosis treated with direct-acting antivirals. J Hepatol.

[388] van der Meer AJ, Feld JJ, Hofer H, Almasio PL, Calvaruso V, Fernandez-Rodriguez CM, Aleman S, Ganne-Carrie N, D’Ambrosio R, Pol S, Trapero-Marugan M, Maan R, Moreno-Otero R (2016). Risk of cirrhosis-related complications in patients with advanced fibrosis following hepatitis C virus eradication. J Hepatol.

[389] Curry MP, Forns X, Chung RT, Terrault NA, Brown R, Fenkel JM, Gordon F, O’Leary J, Kuo A, Schiano T, Everson G, Schiff E, Befeler A (2015). Sofosbuvir and ribavirin prevent recurrence of HCV infection after liver transplantation: an open-label study. Gastroenterology.

[390] Reig M, Marino Z, Perello C, Inarrairaegui M, Ribeiro A, Lens S, Diaz A, Vilana R, Darnell A, Varela M, Sangro B, Calleja JL, Forns X (2016). Unexpected high rate of early tumor recurrence in patients with HCV-related HCC undergoing interferon-free therapy. J Hepatol.

[391] Cardoso H, Vale AM, Rodrigues S, Goncalves R, Albuquerque A, Pereira P, Lopes S, Silva M, Andrade P, Morais R, Coelho R, Macedo G (2016). High incidence of hepatocellular carcinoma following successful interferon-free antiviral therapy for hepatitis C associated cirrhosis. J Hepatol.

[392] Akuta N, Kobayashi M, Suzuki F, Sezaki H, Fujiyama S, Kawamura Y, Hosaka T, Saitoh S, Suzuki Y, Arase Y, Ikeda K, Kumada H (2016). Liver Fibrosis and Body Mass Index Predict Hepatocarcinogenesis following Eradication of Hepatitis C Virus RNA by Direct-Acting Antivirals. Oncology.

[393] Kobayashi M, Suzuki F, Fujiyama S, Kawamura Y, Sezaki H, Hosaka T, Akuta N, Suzuki Y, Saitoh S, Arase Y, Ikeda K, Kumada H (2016). Sustained virologic response by direct antiviral agents reduces the incidence of hepatocellular carcinoma in patients with HCV infection. J Med Virol.

[394] Acar A, Gorenek L, Aydin A, Eyigun CP, Eken A, Sayal A, Pahsa A (2009). Investigation of oxidative stress and antioxidant defense in patients with hepatitis B virus infection and the effect of interferon-alpha plus lamivudine combination therapy on oxidative stress. Mikrobiyol Bul.

[395] Ahmed MM, Abdel-Salam OM, Mohammed NA, Habib DF, Gomaa HE (2013). Oxidative status and the response to pegylated-interferon alpha2a plus ribavirin in chronic genotype 4 HCV hepatitis. EXCLI J.

[396] El-Kannishy G, Arafa M, Abdelaal I, Elarman M, El-Mahdy R (2012). Persistent oxidative stress in patients with chronic active hepatitis-C infection after antiviral therapy failure. Saudi J Gastroenterol.

